# Intramolecular Versus Intermolecular Diels–Alder Reactions: Insights from Molecular Electron Density Theory

**DOI:** 10.3390/molecules30092052

**Published:** 2025-05-05

**Authors:** Luis R. Domingo, Patricia Pérez

**Affiliations:** 1 Independent Researcher, Av. Tirso de Molina 20, 46015 Valencia, Spain; 2 Facultad de Ciencias, Universidad San Sebastián, Campus Ciudad Universitaria, Av. del Condor 720, Ciudad Empresarial, Huechuraba, Santiago 8580704, Chile

**Keywords:** intramolecular Diels–Alder reactions, molecular electron density theory, chemical reactivity, electron localization function, DFT-based reactivity indices, relative interacting atomic energy

## Abstract

The intramolecular Diels–Alder (IMDA) reactions of four substituted deca-1,3,9-trienes and one N-methyleneocta-5,7-dien-1-aminium with different electrophilic/nucleophilic activations have been studied within the Molecular Electron Density Theory (MEDT) and compared to their intermolecular processes. The topological analysis of the electron density and DFT-based reactivity indices reveal that substitution does not modify neither the electronic structure nor the reactivity of the reagents relative to those involved in the intermolecular processes. The analysis of the relative energies establishes that the accelerations found in the polar IMDA reactions follow the same trend as those found in the intermolecular processes. The geometries and the electronic structures of the five transition state structures involved in the IMDA reactions are highly similar to those found in the intermolecular processes. A relative interacting atomic energy (RIAE) analysis of Diels–Alder and IMDA reactions allows for the establishment of the substituent effects on the activation energies. Although the nucleophilic frameworks are destabilized, the electrophilic frameworks are further stabilized, resulting in a reduction in the activation energies. The present MEDT study demonstrates the remarkable electronic and energetic similarity between the intermolecular and intramolecular Diels–Alder reactions. Only the lower, unfavorable activation entropy associated with the latter renders it 10^4^ times faster than the former.

## 1. Introduction

**Scheme 1:**
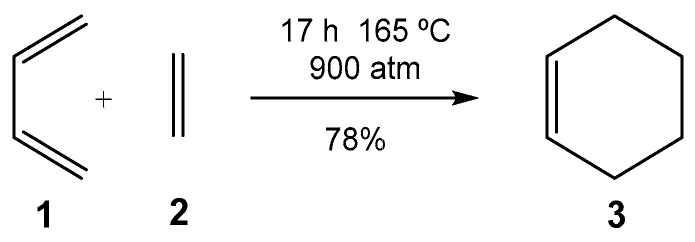
DA reaction between butadiene **1** and ethylene **2**.

Diels–Alder (DA) reactions [1], which belong to the general class of cycloaddition reactions, are among the most useful synthetic reactions in Organic Chemistry as they allow the construction of six-membered carbocyclic compounds with high regio- and stereoselectivity in a single synthetic step [2,3]. They have been extensively studied both experimentally and theoretically. The DA reaction allows the construction of many different types of six-membered cyclic structures by modifying the structure of diene and ethylene. However, not all possibilities are experimentally feasible. Thus, the DA reaction between butadiene **1** and ethylene **2**, which has been selected as the reference for DA reactions in all textbooks [4,5], must be forced to take place, for example, after 17 h at 165 °C and 900 atm, cyclohexene **3** is obtained with a 78% yield (see Scheme 1) [6].

This DA reaction has a high activation energy of 27.5 kcal·mol^−1^ and is strongly exothermic by 40 kcal·mol^−1^ [6]. The unfavorable negative activation entropy associated with this bimolecular process, −40.6 cal·mol^−1^·K^−1^ [7], increases the activation Gibbs free energy to 42.6 kcal·mol^−1^ at 165 °C.

This highly unfavorable activation Gibbs free energy can be reduced in two ways: (i) by diminishing the unfavorable activation enthalpy associated with this DA reaction; and/or (ii) by reducing the negative activation entropy associated with this bimolecular process.

**Figure 1:**
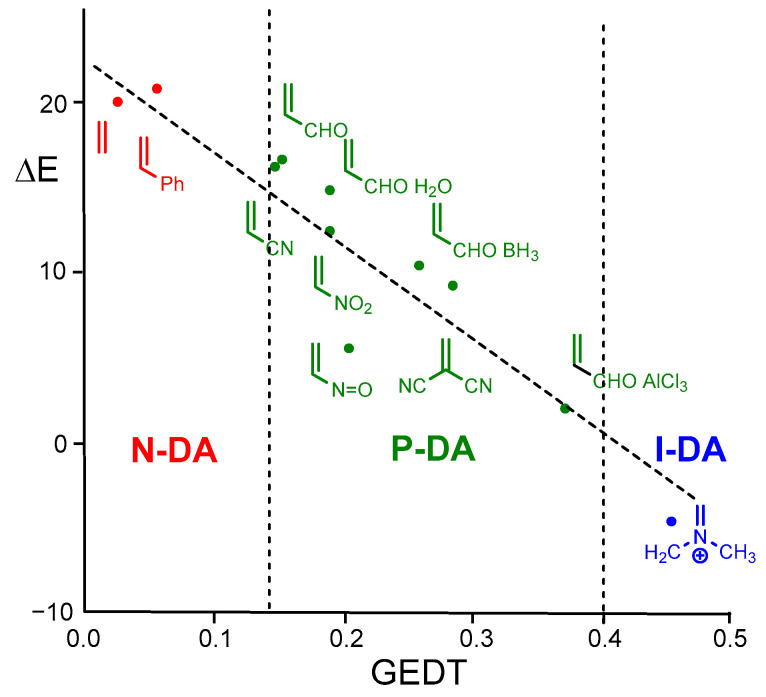
Plot of the activation barriers (ΔE in kcal·mol^−1^) vs. GEDT, in e, *R*^2^ = 0.89, for the DA reactions of Cp **4** with a series of substituted ethylenes of increasing electrophilic character. The classification of the DA reactions, based on GEDT, is included.

A thorough theoretical study of experimental DA reactions has revealed the significant role of global electron density transfer [8] (GEDT) taking place at the transition state structures (TSs) in reducing the activation energies of DA reactions. This discovery enabled the establishment in 2009 of the mechanism for polar DA (P-DA) reactions [9], in which the favorable nucleophilic/electrophilic interactions occurring at the TSs determine the feasibility of the experimental DA reactions [10]. The strong linear correlation found between GEDT and the activation barriers for the DA reactions of Cp **4** with a series of 12 substituted ethylenes of increasing electrophilic character, including an iminium cation, allowed the classification of experimental DA reactions into non-polar DA (N-DA) reactions, which do not proceed easily experimentally, polar DA (P-DA) reactions, and ionic DA (I-DA) reactions, in which one of the reagents is an ionic species (see Figure 1) [9]. Notably, I-DA reactions represent the extreme case of P-DA reactions.

**Scheme 2:**
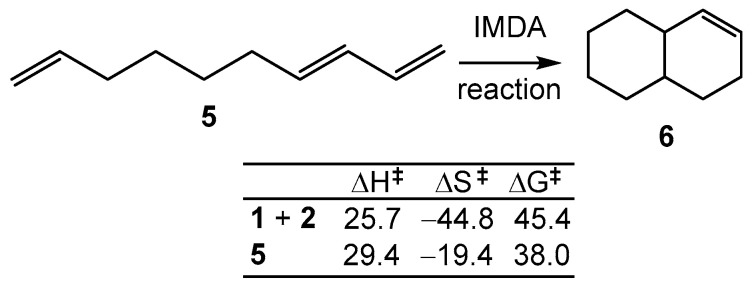
IMDA reaction of (E)-deca-1,3,9-triene **5**. Activation enthalpies and Gibbs free energies are given in kcal·mol^−1^, while activation entropies are given in cal·mol^−1^·K^−1^. The data corresponding to the DA reaction between butadiene **1** and ethylene **2** are included (see Scheme 1).

On the other hand, the intramolecular processes allow for the reduction of the unfavorable negative activation entropy associated with bimolecular processes. Scheme 2 shows the thermodynamic data for the N-DA reaction between butadiene **1** and ethylene **2** shown in Scheme 1, along with those for the intramolecular Diels–Alder (IMDA) reaction of (E)-deca-1,3,9-triene (DTE) **5 [11]**. The B3LYP/6-311G(d,p) activation enthalpy for the N-DA reaction of **1** with **2**, ΔH^≠^ = 25.7 kcal·mol^−1^, and the computed activation entropy, ΔS^≠^ = −44.8 cal·mol^−1^·K^−1^, were close to the experimental values, 27.5 kcal·mol^−1^ and −40.6 cal·mol^−1^·K^−1^, respectively [6,7]. These high values raise the activation Gibbs free energy to 45.4 kcal·mol^−1^ at 165 °C (see Scheme 2). Despite the fact that the computed activation enthalpy associated with the IMDA reaction of **5**, ΔH^≠^ = 29.4 kcal·mol^−1^, is higher than that associated with the intermolecular one, the lower activation entropy associated with this intramolecular process, ΔS^≠^ = −19.4 cal·mol^−1^·K^−1^, reduces the activation Gibbs free energy to 38.0 kcal·mol^−1^. This behavior explains the feasibility of non-polar IMDA reactions.

IMDA reactions permit contiguous cycles to be created in a single synthetic step [12] with remarkable stereoselectivity [13,14,15]. These reactions are widely used as synthetic routes in the total synthesis of natural products [13,14,15,16,17,18].

**Scheme 3:**
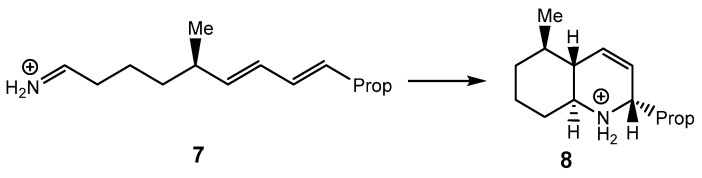
Ionic IMDA reaction of dieniminium cation **7**.

IMDA reactions have been extensively studied from a theoretical point of view [11,19,20,21,22,23,24]. The ionic IMDA reaction of dieniminium cation **7 [11]**, experimentally reported by Grieco and Parke [24], was recently studied within the framework of Molecular Electron Density Theory [25] (MEDT) (Scheme 3). The activation enthalpy associated with this IMDA reaction, 8.7 kcal·mol^−1^, was close to that of the I-DA reaction between butadiene **1** and ethaniminium **9**, 9.3 kcal·mol^−1^. However, the intermolecular I-DA reaction showed a higher activation Gibbs free energy, 25.3 kcal·mol^−1^, than the intramolecular one, 12.6 kcal·mol^−1^, as a consequence of the lower negative activation entropy associated with the latter process, −14.3 cal·mol^−1^·K^−1^ [11]. This ionic IMDA reaction exhibits complete *re*/*exo* and *si*/*endo* diastereoselectivity, which is controlled by the most favorable chair conformations adopted by the –(CH_2_)_4_- chain linking the butadiene and iminium frameworks (see dieniminium **7** in Scheme 3).

**Chart 1:**
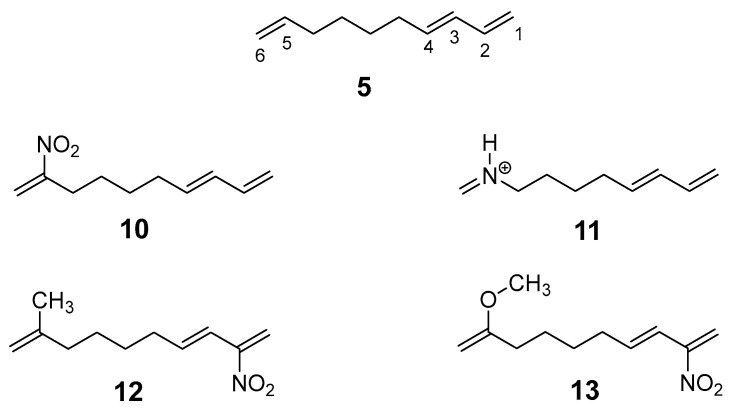
DTEs **5**, **10**, **12** and **13** and MODA **11** used in the study of the N-IMDA, P-IMDA, and I-IMDA reactions.

**Chart 2:**
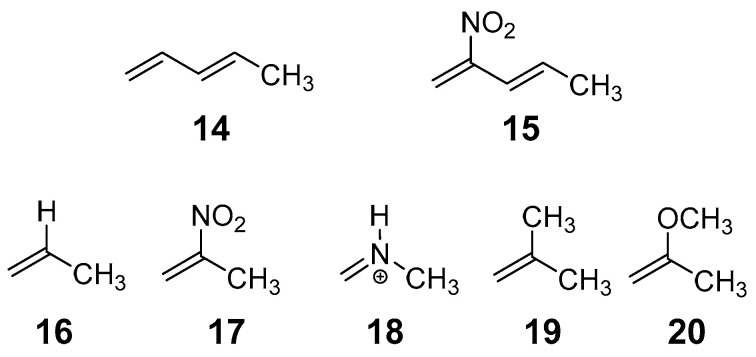
Dienes, **14** and **15**, and ethylenes, **16**–**20**, involved in the studied DA reactions.

Due to the relevance of the IMDA reactions in organic synthesis, the similarities between the non-polar (N-IMDA), polar (P-IMDA) and ionic (I-IMDA) IMDA reactions of DTEs **5, 10**, **12** and **13** and N-methyleneocta-5,7-dien-1-aminium (MODA) **11** shown in Chart 1, with the N-DA, P-DA and I-DA reactions of pentadienes, **14** and **15**, with the ethylenes, **16**–**20**, shown in Chart 2, are studied herein within the MEDT at the M06-2X/6-311G(d,p) computational level. The complete study of the intermolecular DA reactions between the reagents shown in Chart 2 is reported in the Supplementary Materials.

## 2. Results and Discussion

This MEDT study is divided into five sections: (i) initially, the topological analysis of the electron localization function (ELF) of the electronic structure of the four DTEs **5**, **10**, **12** and MODA **11** is conducted, along with an analysis of the chemical properties of the reagent ground states (GSs); (ii) second, the potential energy surfaces (PES) of the IMDA reactions of DTEs **5**, **10**, **12** and **13** and MODA **11** are explored; (iii) third, an ELF topological analysis is achieved on the electronic structure of the five TSs involved in the IMDA reactions; (iv) fourth, a comparative analysis of the kinetics of the IMDA reactions relative to the kinetics of the DA reactions is performed; and finally, (v) the fifth section focuses on an RIAE analysis of the DA reactions of **14** with **16** or **17**, and that of **15** with **20**, as well as the IMDA reactions of the DTEs **5**, **10** and **13**.

### 2.1. Study of the Electronic Structure and Chemical Properties of the DTEs **5**, **10**, **12** and **13** and MODA **11** in the GS

#### 2.1.1. Study of the Electronic Structure of the DTEs **5**, **10**, **12** and **13** and MODA **11**

**Figure 2:**
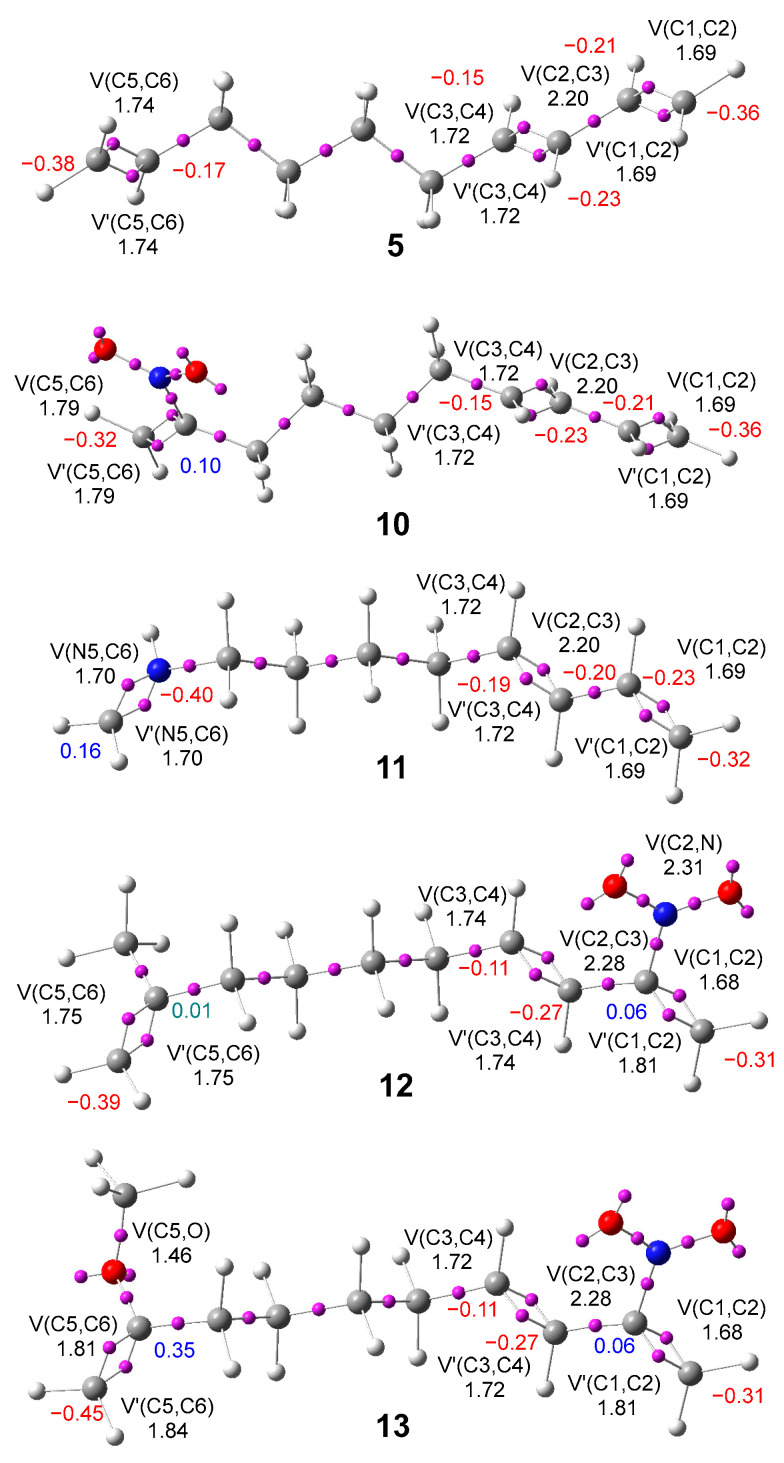
ELF basin attractor positions and the populations of the most relevant valence basins of DTEs **5**, **10**, **12**, and **13** and MODA **11**, calculated at the M06-2X/6-311G(d,p) level in the gas phase. Natural atomic charges are reported as the average number of electrons, e. Negative charges are highlighted in red, positive charges in blue, and negligible charges in green.

Before exploring the PES of the IMDA reactions shown in Chart 1, the electronic structures at the GS of DTEs **5**, **10**, **12** and **13** and MODA **11** were investigated through a topological analysis of the ELF [26]. ELF provides a quantitative characterization of the electron density distribution in a molecule, facilitating a correlation between its electronic structure and reactivity. The attractor positions of the ELF basins, the populations of the most relevant valence basins and the natural atomic charges of the DTEs **5**, **10**, **12** and **13** and MODA **11**, computed at the M06-2X/6-311G(d,p) level, are shown in Figure 2. ELF of the compounds **14**–**20**, involved in the DA reaction, is analyzed in Section 1 in the Supplementary Materials.

ELF topological analysis of DTE **5** shows the presence of two disynaptic basins, V(C1,C2) and V’(C1,C2), integrating a total of 3.38 e, associated with a depopulated C1–C2 double bond, one V(C2,C3) disynaptic basin integrating 2.20 e, associated with a populated C2–C3 single bond, and two disynaptic basins, V(C3,C4) and V’(C3,C4), integrating a total of 3.44 e, associated with a depopulated C3–C4 double bond, characterizing the conjugated 1,3-butadiene system of DTE **5**. On the other hand, the C5–C6 bonding region is characterized by the presence of two disynaptic basins, V(C5,C6) and V’(C5,C6), integrating a total of 3.48 e, associated with a depopulated C5–C6 double bond. The ELF of the diene and ethylene frameworks of **5** is very similar to that of pentadiene **14** and propene **16**, as shown in Figure S1 in the Supplementary Materials.

The C1=C2–C3=C4 frameworks of 8-nitro-deca-1,3,9-triene **10** and the MODA cation **11** have ELF topological behaviors closely resembling that of DTE **5** (See Figure 2). The presence of the electron-withdrawing (EW) NO_2_ group on the C5 carbon of **10** causes a slight increase in the population of the C5–C6 bonding region, which is characterized by the presence of two disynaptic basins, V(C5,C6) and V’(C5,C6), integrating a total of 3.58 e. On the other hand, the N5–C6 bonding region of MODA cation **11** shows only a slight depopulation of 0.18 e compared to C5–C6 region in the nitro derivative DTE **10**. The ELF of the ethylene frameworks of the DTE **10** and the MODA cation **11** are very similar to those of 2-nitro-propene **17** and iminium cation **18**, respectively, as shown in Figure S1 in the Supplementary Materials.

Finally, the presence of the EW NO_2_ group on the C2 carbon of DTEs **12** and **13** induces negligible changes in the C1=C2–C3=C4 bonding region compared to DTE **5**. Only the population of V(C2,C3) disynaptic basins are increased by 0.08 e. On the other hand, while the presence of an electron-releasing (ER) CH_3_ group decreases the population of the C5–C6 bonding region in compound **12**, the presence of the strong ER OCH_3_ group on the C5 carbon of compound **13** increases the population of the C5–C6 bonding region by 0.17 e. The ELF of the butadiene frameworks of the DTEs **12** and **13** are very similar to that of 2-nitro-pentadiene **15**, while the ethylene frameworks are very similar to those of propene **16** and 2-methoxy-propene **20**, respectively (see Figure S1 in the Supplementary Materials).

Thus, the present ELF analysis of DTEs **5**, **10**, **12** and **13** and MODA **11** shows a strong similarity between the electronic structure of the diene and ethylene frameworks of these compounds and those of compounds **14**–**20**, which are involved in the selected DA reaction.

The natural atomic charges [27,28] of the most relevant centers of DTEs **5**, **10**, **12** and **13** and MODA **11** are presented in Figure 2. In general, all carbon atoms belonging to the conjugated double bond framework are negatively charged between −0.11 and −0.45 e. The negative charge at the non-substituted carbon atoms of these DTEs depends on the number of hydrogen atoms attached to them; this behavior is caused by the higher electronegativity of the carbon atom compared to the hydrogen atom.

The presence of the NO_2_, CH_3_ and OCH_3_ groups in DTEs **10**, **12** and **13** causes a significant change in the charge on the carbon atom to which they are attached. Thus, the presence of the CH_3_ groups at **12** makes the C5 carbon negligibly charged, while the presence of the NO_2_ and OCH_3_ groups makes the C2 or C5 carbons to which they are attached positively charged (see Figure 2). This behavior is a consequence of the higher electronegative character of the N nitrogen or O oxygen atoms compared to the C carbon one, rather than the EW or ER character of the corresponding groups. Interestingly, the MODA cation **11**, formed by protonation of the N5 nitrogen in the corresponding imine, is negatively charged by −0.40 e.

A comparative analysis of the natural atomic charges of DTEs **5**, **10**, **12** and **13** and MODA **11** with those of compounds **14**–**20** involved in the selected DA reaction, shows a strong similarity between them (see Figure S1 in the Supplementary Materials).

The present structural analysis of the DTEs **10**, **12** and **13** shows that the inclusion of the EW or ER groups on the unsaturated frameworks of these compounds does not substantially modify the electronic structures of these DTEs relative to that of DTE **5**. Only the charges of the C2 or C5 carbon atoms to which the activating groups are attached are markedly modified. 

#### 2.1.2. Analysis of the Chemical Properties of DTEs **5**, **10**, **12** and **13** and MODA **11**

A useful method for comprehending reactivity in polar reactions is the analysis of the DFT-based reactivity indices at the GS of the reagents [29,30]. Following, the DFT-based reactivity indices of DTEs **5**, **10**, **12** and **13** and MODA **11** were analyzed to understand their chemical reactivity. The DFT-based reactivity indices were computed at the B3LYP/6-31G(d) computational level, as it was used to establish electrophilicity and nucleophilicity scales [30]. The reactivity indices of the cations MODA **11** and iminium **19** were computed in DMSO [31]. Table 1 summarizes the B3LYP/6-31G(d) global reactivity indices, including electronic chemical potential μ, chemical hardness η, as well as the electrophilicity ω and nucleophilicity *N* indices of DTEs **5**, **10**, **12** and **13** and MODA **11**. The reactivity indices of the butadiene and ethylene derivatives **14**–**20** are given in Table S1 in the Supplementary Materials.

Previous studies of IMDA reactions have shown that analyzing the electrophilicity [32] ω and nucleophilicity [33] *N* indices of the GS reagents provides valuable information about the electrophilicity and nucleophilicity of the butadiene and ethylene frameworks of these complex molecules [11].

The values of the electrophilicity ω and nucleophilicity *N* indices of DTE **5**, 0.93 and 3.19 eV, respectively, allow its classification as a moderate electrophile and a strong nucleophile [30]. The electrophilicity ω and nucleophilicity *N* indices of 1,3-pentadiene **14** are 0.91 and 3.20 eV, respectively, and those of propene **16** are 0.60 and 2.32 eV (see Table S1 in the Supplementary Materials). A comparison of these values with those of DTE **5** indicates that its nucleophilicity *N* index is associated with the diene framework. The very low electrophilicity ω index of propene **16**, ω = 0.60 eV, indicates that the IMDA reaction of DTE **5** will have a non-polar character, being classified as null electron density flux (NEDF) [34].

The values of the electrophilicity ω and nucleophilicity *N* indices of 9-nitro-deca-1,3,9-triene **10**, 2.49 and 3.09 eV, respectively, allow its classification as a strong electrophile and a strong nucleophile. While the electrophilicity ω index is closer to that of 2-nitro-propene **17**, ω = 2.43 eV, the nucleophilicity *N* index is closer to that of 1,3-pentadiene **14** (see Table S1 in the Supplementary Materials). The analysis of the reactivity indices indicates that along the P-IMDA reaction of DTE **10**, the flux of the electron density will occur from the butadiene framework to the ethylene one, in a reaction of forward electron density flux (FEDF) [34].

The values of the electrophilicity ω and nucleophilicity *N* indices in DMSO of MODA cation **11**, 3.00 and 2.64 eV, respectively, allow its classification as a strong electrophile and a strong nucleophile [30]. The electrophilicity ω index of MODA cation **11** is closer to that of iminium cation **18**, ω = 2.91 eV (see Table S1 in the Supplementary Materials). Consequently, along the I-IMDA reaction of MODA cation **11**, the electron density flux will take place from the butadiene framework to the protonate iminium one, in a reaction of FEDF.

The values of the electrophilicity ω and nucleophilicity *N* indices of 2-nitro-9-methyl-deca-1,3,9-triene **12**, 2.38 and 2.54 eV, respectively, allow its classification as a strong electrophile and a moderate nucleophile. The electrophilicity ω index of this species is closer to that of 2-nitro-pentadiene **15**, ω = 2.43 eV, while the nucleophilic *N* index is closer to that of isobutene **19**, *N* = 2.60 eV. Consequently, along the P-IMDA reaction of DTE **12**, the electron density flux will take place from the ethylene framework to the butadiene one, in a reaction of reverse electron density flux, REDF.

Finally, the values of the electrophilicity ω and nucleophilicity *N* indices of 2-nitro-9-methoxy-deca-1,3,9-triene **13**, 2.43 and 2.97 eV, respectively, allow its classification as a strong electrophile and borderline of a strong nucleophile. The nucleophilicity *N* index of this species is lower than that of 2-methoxy-propene **20**, *N* = 3.24 eV. Consequently, along the P-IMDA reaction of DTE **13**, the electron density flux will take place from the ethylene framework to the butadiene one, in a reaction of REDF.

In P-DA reactions involving non-symmetric reagents, the most favorable reaction path is that involving the two-center interaction between the most nucleophilic and the most electrophilic centers of the reagents. In this sense, the analysis of the electrophilic $\;P_{k}^{+}\;$ and nucleophilic $P_{k}^{-}$ Parr functions [35] has become a powerful tool to study regioselectivity in P-DA and P-IMDA reactions.

**Chart 3:**
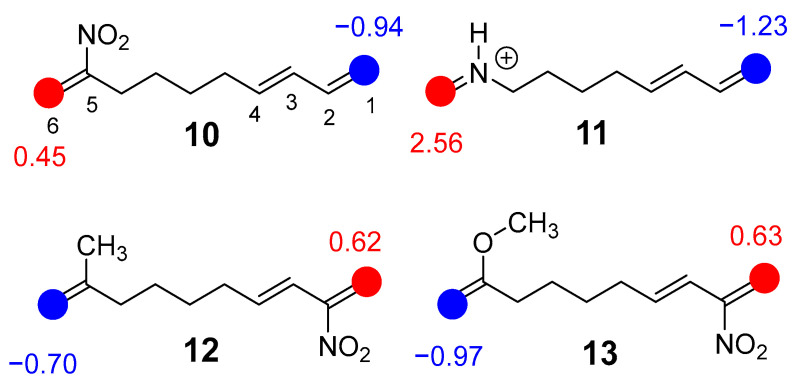
Most electrophilic, in red, and nucleophilic, in blue, centers, together with the corresponding R_k_ values of DTEs **10**, **12**, and **13** and MODA **11**.

In 2012, Chattaraj et al. proposed a local reactivity difference, R_k_, index to analyze the local electrophilic and/or nucleophilic activation within a complex organic molecule [36]. Together with the electrophilic and/or nucleophilic behavior of the center k given by the sign, the magnitude of the R_k_ index accounts for the extent of the electronic activation. The R_k_ index has become a powerful tool to analyze the electrophilic and nucleophilic center in a molecule, allowing prediction of the reactivity in an intramolecular polar reaction [36]. The electrophilic $P_{k}^{+}\;$ and nucleophilic $P_{k}^{-}$ Parr functions, together with the local reactivity difference R_k_ indices of DTEs **10**, **12** and **13** and MODA **11**, involved in the P-IMDA and I-IMDA reactions, are given in Table 2. Chart 3 shows a graphical representation of the most electrophilic, lowest R_k_ < 0, and nucleophilic, highest R_k_ > 0, center of DTEs **10**–**12**.

The local electrophilic/nucleophilic activation in the butadiene/ethylene system of these DTEs depends on the position of the EW/ER substituents in these unsaturated systems. Thus, the position of the substituent in the butadiene or ethylene framework determines the formation of a mixture of regioisomeric bicyclic compounds in P-IMDA reactions. In the present MEDT study, the selected mono- or disubstitution on the C2 or C5 positions of the deca-1,3,9-triene system, as shown in Chart 1, activates the C1 and C6 positions, respectively, allowing the most favorable electrophilic/nucleophilic interaction along these P-IMDA reactions (see later).

### 2.2. Study of the IMDA Reactions of DTEs **5**, **10**, **12** and **13** and MODA **11**

**Scheme 4:**
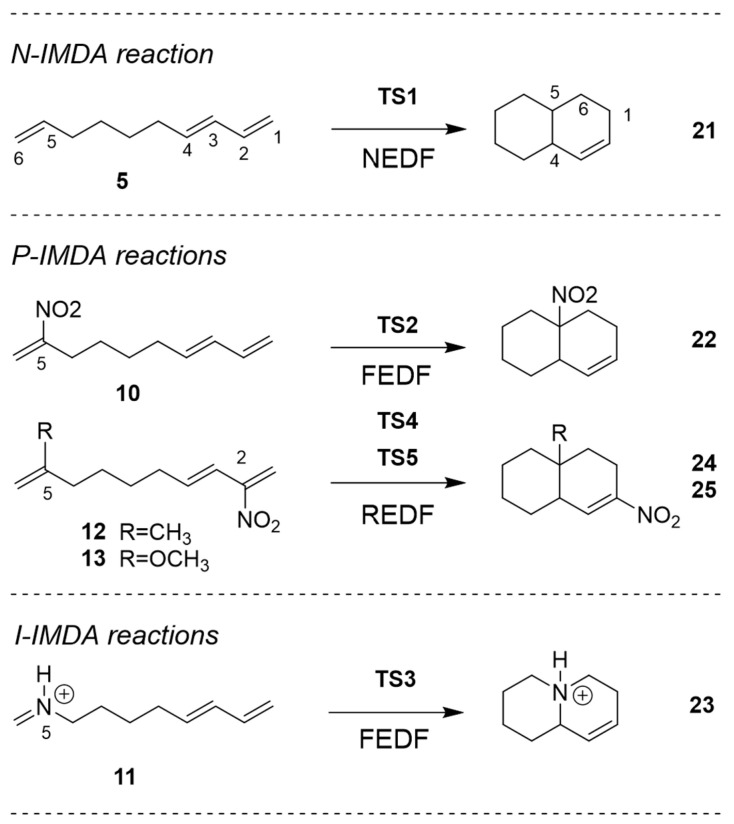
N-IMDA of **5**, P-IMDA of **10**, **12** and **13**, and I-IMDA of **11** reactions.

Then, the PESs of the most favorable reaction paths associated with the IMDA reactions of DTEs **5**, **10**, **12** and **13** and MODA **11** were studied (see Scheme 4). Along these reaction paths, the -(CH_2_)_4_- chain adopts a chair conformation, diminishing the strain associated with these intramolecular processes [11]. Along each IMDA reaction, one reagent, one TS, and one bicyclic compound were located and characterized, indicating that these IMDA reactions take place through a one-step mechanism. The M06-2X/6-311G(d,p) gas phase relative energies of the stationary points involved in the IMDA reactions of DTEs **5**, **10**, **12**, and **13** and MODA **11** are given in Table 3, while the gas phase total energies are given in Table S3 in the Supplementary Materials. The M06-2X/6-311G(d,p) gas phase total and relative energies of the stationary points involved in the DA reactions of compounds **14**–**20** are given in Table S2 in the Supplementary Materials. The total and relative electronic energies in tetrahydrofuran (THF) are given in Tables S4 and S5 in the Supplementary Materials.

Some appealing conclusions can be obtained from the relative energies given in Table 3: (i) the five IMDA reactions are strongly exothermic, ranging from −43.8 (**5**) to −52.2 (**11**) kcal·mol^−1^. Consequently, these IMDA reactions can be considered irreversible and kinetically controlled; (ii) the relative energies of the TSs range from 23.9 kcal·mol^−1^ for the N-IMDA reaction of DTE **5** to −0.8 kcal·mol^−1^ for the I-IMDA reaction of MODA cation **11**; (iii) these relative energies indicate that the substitution on the DTE system has a greater impact on the kinetics than on the thermodynamics of the reactions; (iv) **TS3** is located 0.8 kcal·mol^−1^ below the extended conformation of the DTE system, which has been selected as the energy reference for these IMDA reactions. However, when the PES is calculated in THF, the relative energy becomes positive by 8.3 kcal·mol^−1^ due to the strong stabilization of the MODA cation **11** (see Table S4 in the Supplementary Materials); and finally, (v) in the REDF P-IMDA reactions of DTEs **12** and **13**, the double substitution at the diene and ethylene frameworks is required to increase the nucleophilic character of the ethylene framework and change the nucleophilic character of the butadiene system of DTE **5** to an electrophilic one.

A comparison of the relative energies of the TSs and cycloadducts of the IMDA reactions of DTEs **5**, **10**, **12** and **13** and MODA **11**, given in Table 3, with those of the DA reactions of compounds **14**–**20**, given in Table S2, provides important insight into the IMDA reactions to be obtained: (i) the series of five IMDA and DA reactions exhibit a similar strong exothermic character; (ii) the exothermic character of the IMDA reactions is 2 kcal·mol^−1^ lower than that of the DA reactions due to the presence of a lower strain associated with the formation of the bicyclic systems; (iii) the relative energies of the TSs associated with the intermolecular DA reactions are between 2.9 (**5**) and 9.1 (**11**) kcal·mol^−1^ lower than those associated with the IMDA reaction; (iv) these energy differences increase as the activation energies decrease, i.e., the more polar the reactions, the greater difference, and finally (v) in spite of this difference, the same trend in the kinetics of the reactions is found in both series of reactions with the substitution on the butadiene and ethylene systems influencing the reactivity.

**Figure 3:**
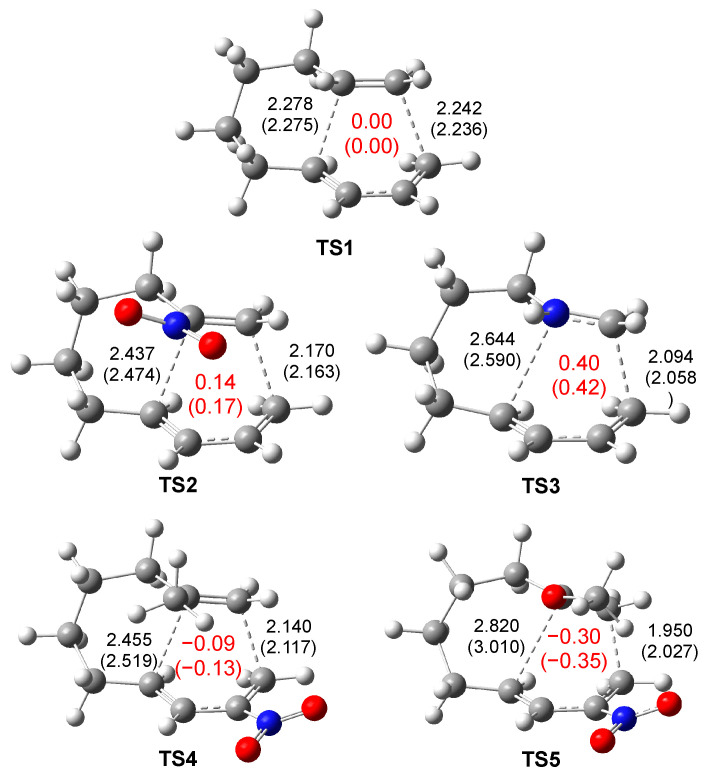
M06-2X/6-311G(d,p) gas phase optimized geometries of **TS1**–**TS5** associated with the IMDA reactions of DTEs **5**, **10**, **12** and **13** and MODA **11**. GEDT values are shown in red and expressed as the average number of electrons, e. Bond lengths are given in Angstroms. Values in THF are given in parentheses.

The gas phase geometries of the TSs involved in the IMDA reactions of DTEs **5**, **10**, **12** and **13** and MODA **11** are given in Figure 3. In all TSs, the -(CH_2_)_4_- chain that links the butadiene and ethylene frameworks adopts a chair conformation, reducing the strain energy associated with these intramolecular processes. Note that at GS of the reagents, the -(CH_2_)_4_- chain adopts an extended *anti*-conformation.

The C1–C6 distances at the TSs range from 1.95 to 2.24 Å, while the C4–C5 distances are in the range from 2.28 to 2.80 Å. While **TS1** corresponds to a synchronous C–C single bond formation process, the other four TSs correspond to asynchronous single bond formation processes in which the shorter distance is between the C1 and C6 carbons. This behavior agrees with the analysis of the reactivity difference R_k_ indices at the GS of the reagents, which indicates that the C1 and C6 carbons are the most nucleophilic/electrophilic centers of these molecules.

For the P-IMDA and I-IMDA reactions, the asynchronicity increases with the increase in the polar character of the reactions (see later). While the C1–C6 distances between the two pairs of interacting atoms decrease with the increase in nucleophilic/electrophilic interactions, the C4–C5 distances increase, i.e., the more polar the reaction, the more asynchronous the TS.

The inclusion of solvent effects of THF in geometrical optimizations does not significantly modify the gas phase geometries (see Figure 3).

Analysis of the GEDT [8] at the TSs involved in these IMDA reactions enables the quantification of the polar character of these cycloaddition reactions. The GEDT values calculated at the five TSs are presented in Figure 3. GEDT values below 0.05 e indicate non-polar processes, while values above 0.20 e denote highly polar processes. Additionally, the sign of the GEDT at the TSs clearly distinguishes the polar DA reactions as FEDF, with GEDT > +0.05 e, or REDF, with GEDT < −0.05 e. Non-polar 32CA reactions, characterized by a negligible GEDT (≤|0.05| e), are classified as NEDF [34].

While the GEDT value at **TS1**, 0.00 e, indicates that it is associated with an N-IMDA reaction classified as NEDF, **TS3**, associated with an I-IMDA reaction, displays the maximum GEDT value, 0.40 e. The positive GEDT values found at **TS2** and **TS3** classify these P-IMDA reactions as FEDF. Finally, the negative GEDT values found at **TS4** and **TS5** allow them to be classified as REDF. **TS5**, with GEDT = −0.30 e, corresponds to the most asynchronous TS of the three polar ones. The inclusion of THF solvent effects slightly increases the GEDT at the polar TSs, favoring the electron density transfer process (see Figure 3).

**Figure 4:**
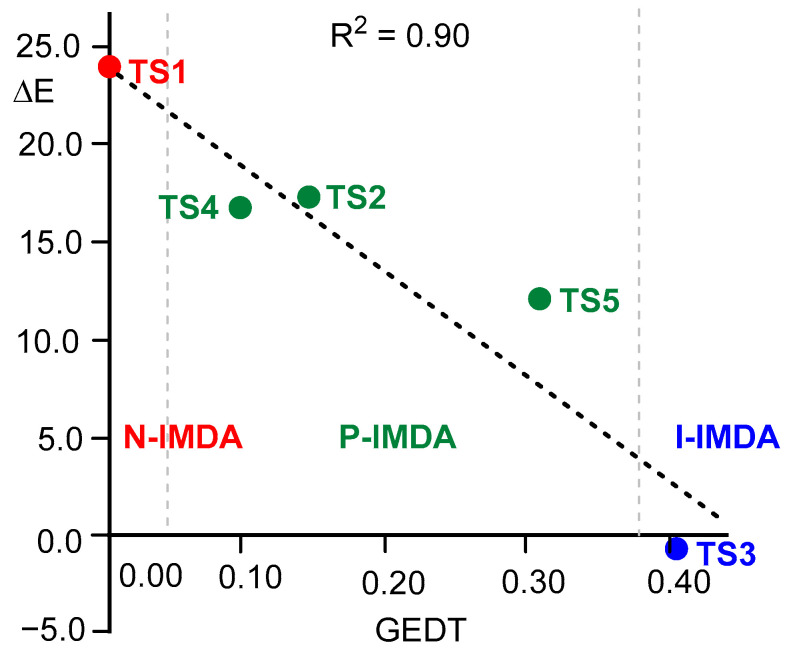
Plot of the relative energies, ΔE in kcal·mol^−1^, of TSs of the IMDA reactions vs. the GEDT expressed as the average number of electrons e, computed at the corresponding TSs. The three groups of N-IMDA, P-IMDA, and I-IMDA reactions are shown in red, green and blue, respectively.

Recently, the crucial role of GEDT in TSs of P-DA reactions in reducing activation energies has been established [10], a behavior that constitutes the foundation of P-DA reactions, as first proposed in 2009 [9]. Figure 4 shows the plot of the relative energies of the TSs of five IMDA reactions vs. the GEDT computed at the corresponding TSs. As can be seen, a good linear correlation, R^2^ = 0.90, is obtained: the higher the GEDT, the faster the reaction. This graph, which is similar to that obtained for intermolecular DA reactions (see Figure 1), clearly differentiates N-IMDA, in red, P-IMDA, in green, and I-IMDA, in blue, reactions (see Figure 4).

### 2.3. ELF Topological Analysis of the Electronic Structure of **TS1**–**TS5**

**Figure 5:**
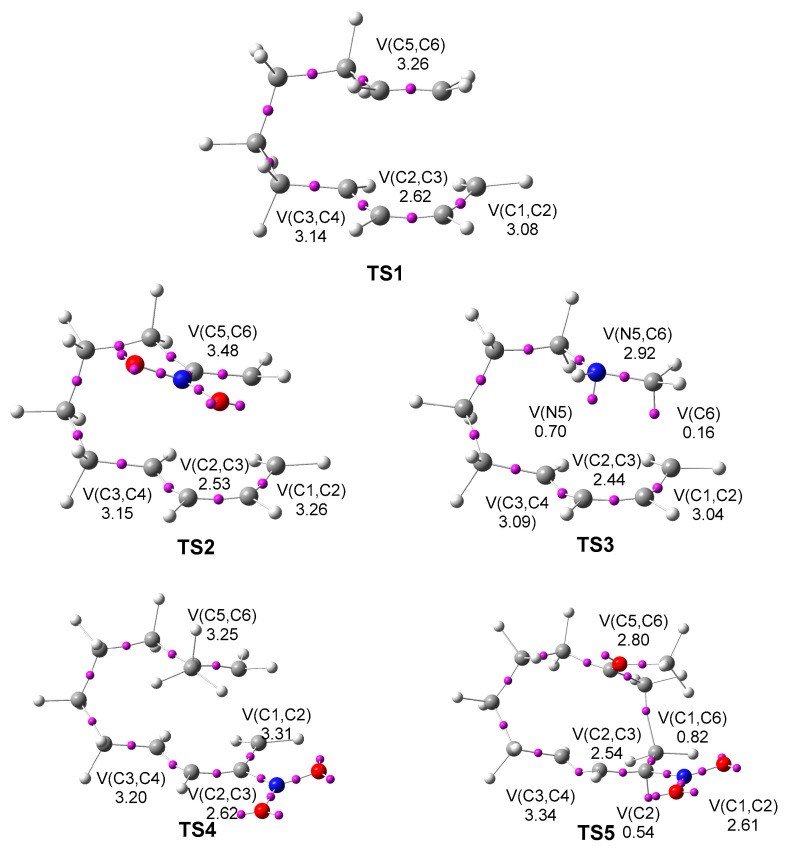
ELF basin attractor positions and the populations of the most relevant valence basins of **TS1**–**TS5**, calculated at the M06-2X/6-311G(d,p) level in the gas phase.

Next, an ELF topological analysis was subsequently conducted on the **TS1**–**TS5** associated with the IMDA reactions. The attractor positions of the ELF basins and key valence basin populations for the five TSs are given in Figure 5.

The five TSs show the depopulation of the C1–C2, C3–C4 and C5–C6 bonding regions, which are characterized by the presence of only one V(C1,C2), V(C3,C4) and V(C5,C6) disynaptic basin, integrating less than 3.48 e. Note that these bonding regions in DTEs **5**, **10**, **12** and **13** and MODA **11** are characterized by the presence of two disynaptic basin, V(Cx,Cy) and V’(Cx,Cy), integrating more than 3.50 e. Together with these depopulations, the C2–C3 bonding regions are slightly populated, as shown by the presence of one V(C2,C3) disynaptic basin integrating more than 2.53 e. Note that at the final cycloadducts, the C2–C3 bonding regions become C2–C3 double bonds.

**TS3** also shows the presence of two new V(N5) and V(C6) monosynaptic basins integrating 0.70 and 0.16 e, respectively. The electron density of these new monosynaptic basins comes from the depopulation of the N5–C6 bonding region in MODA cation **11**, as well as from the high GEDT taking place at **TS3**. On the other hand, **TS5** shows the presence of a new V(C1,C6) disynaptic basin integrating 0.82 e, indicating that the formation of the new C1–C6 single bond has already begun at this TS.

The present ELF topological analysis indicates that while the more energetic **TS1**, **TS2**, and **TS4** are more delayed, the more favorable **TS3** and **TS5** are more advanced, i.e., the easier it is to reach the TS, the more advanced the reaction.

An IRC analysis of the highly asynchronous **TS5** indicates that this P-IMDA reaction is associated with a *two-stage one-step* mechanism [37] in which the formation of the C4–C5 single bond begins when the formation of the C1–C6 single bond is practically completed.

### 2.4. Comparative Analysis of the Kinetics of the IMDA Reactions with Respect to the Kinetics of the DA Reactions

Next, the relative reaction rate constants (RRRCs), k_r_, computed using the Eyring-Polanyi equation [38], for the DA reactions of compounds **14**–**20** and the IMDA reactions of DTEs **5**, **10**, **12**, and **13** and MODA **11** were analyzed. To this end, all gas phase stationary points involved in these intermolecular and intramolecular DA reactions were optimized in THF, and then the thermodynamic data were obtained by frequency calculations carried out in THF at 66 °C. The electronic energies and the thermodynamic data of the stationary points involved in the DA reactions of compounds **14**–**20**, and the IMDA reactions of DTEs **5**, **10**, **12** and **13** and MODA **11**, are given in Tables S4 and S5, respectively, in the Supplementary Materials. Table 4 summarizes the calculated activation Gibbs free energies for **TS1**–**TS10**. Additionally, this table includes the RRRCs, $k_{r}^{I}$, with respect to the P-DA reaction between pentadiene **14** and 2-nitropropene **17**, via **TS7**, and with respect to the P-IMDA reaction of the 9-nitro derivative DTE **10**, via **TS2**. On the other hand, $k_{r}^{2}$, represents the RRRCs of each IMDA reaction with respect to the corresponding intermolecular DA reaction.

The RRRCs $k_{r}^{I}$ for the DA reactions with respect to the P-DA reaction between pentadiene **14** and 2-nitropropene **17** via **TS7** range from 1.5 × 10^−7^ for the N-DA reaction between pentadiene **14** and propene **16** via **TS6** to 1.1 × 10^6^ for I-DA reaction between **14** and iminium cation **18** via **TS8**. On the other hand, the RRRCs $k_{r}^{I}$ for the IMDA reactions with respect to the P-IMDA reaction of the 9-nitro derivative DTE **10** via **TS2** range from 5.6 × 10^−5^ for the N-IMDA reaction of DTE **5** via **TS1** to 1.2 × 10^7^ for the I-IMDA reaction of MODA cation **11** via **TS3**.

Some appealing conclusions can be drawn from the kinetic data given in Table 4: (i) the N-DA and N-IMDA reactions involving non-substituted unsaturated compounds are highly unfavorable; the N-DA reaction between pentadiene **14** and propene **16** via **TS6** is 1.5 × 10^−7^ times slower than the P-DA reaction between **14** and 2-nitropropene **17** via **TS7**. Despite the unfavorable character of the N-IMDA reaction of DTE **5**, this reaction is 3.0 × 10^5^ times faster than the N-DA between pentadiene **14** and propene **16**; (ii) the I-IMDA of MODA cation **11** via **TS3** is 1.2 × 10^7^ times faster than the P-IMDA reaction of the 9-nitro derivative DTE **10** via **TS2**, as a consequence of the higher GEDT taking place at the ionic process (see Figure 4); (iii) the double substitution in the P-IMDA reaction of the 2-nitro-9-methoxy derivative DTE **13** via **TS5** is 7.9 × 10^4^ times faster than the P-IMDA reaction of 9-nitro derivative DTE **10** via **TS2**, again in agreement with the higher polar character of the former; and finally, (iv) a similar trend is observed for the intermolecular P-DA and I-DA reactions, indicating that the substitution on the unsaturated compounds involved in the DA and IMDA reactions causes similar kinetic effects (see Table 4).

The present comparative analysis of the kinetic parameters of the five DA and IMDA reactions shows that the substitution on the unsaturated compounds involved in these cycloaddition reactions produces a similar effect on the kinetics of these reactions. However, the IMDA reactions are approximately 10^4^ times faster than the intermolecular DA reactions because of the less unfavorable activation entropies associated with the intramolecular processes (see Tables S3 and S4 in the Supplementary Materials). This behavior reduces the activation Gibb free energies associated with the intramolecular reaction by 4.5 and 8.5 kcal·mol^−1^ compared to the intermolecular ones (see Table 4).

### 2.5. Comparative RIAE Analysis of the DA Reactions of **14** with **16** or **17**, and That of **15** with **20**, and the IMDA Reactions of the DTEs **5**, **10** and **13**

Finally, to determine the electronic effects of the substituents on the butadiene or ethylene frameworks of the DA reactions of pentadiene **14** with propene **16** or 2-nitropropene **17**, and those of 2-nitropentadiene **15** with vinyl ether **20**, as well as the IMDA reactions of DTE **5**, 9-nitro derivative DTE **10**, and the 9-methyl-2-nitro derivative DTE **13** in the activation energies, a comparative relative interacting atomic energy [39] (RIAE) analysis of the corresponding TSs was performed. The RIAE analysis provides a rationalization of the energy costs associated with the changes in electron density at the TSs, on which MEDT is based [25]. The theoretical background of the RIAE is given in Supplementary Materials. The M06-2X/6-311G(d,p) gas-phase ξ$E_{total}^{X}$ total, ξ$E_{intra}^{X}$ intra-atomic, and ξ$E_{inter}^{X}$ interatomic energies of the butadiene, ethylene and the –(CH_2_)_4_– chain frameworks of the TSs are given in Table 5.

A recent RIAE study the P-DA reaction of cyclopentadiene **4** with the series of cyanoethylenes **26** has shown that the electronic stabilization of the electrophilic framework, resulting from GEDT in P-DA reactions, is the main factor responsible for the reduction in the activation energies [10]. In polar processes, although the nucleophilic framework is destabilized by the loss of electron density, the greater stabilization of the electrophilic framework upon receiving the electron density is responsible for the reduction in activation energies [10].

The RIAE analysis of **TS6** associated with the N-DA reaction of pentadiene **14** with propene **16** shows that the destabilization of both the ethylene framework, ξ$E_{total}^{Et}$ = 12.4 kcal·mol^−1^, and the butadiene framework, ξ$E_{total}^{Bu}$ = 8.5 kcal·mol^−1^, are responsible for the high RIAE activation energies, ξ$E_{total}^{Bu+Et}$ = 20.9 kcal·mol^−1^, associated with this non-polar reaction. Although the ξ$E_{inter}^{X}$ interatomic energies are stabilizing at the two frameworks, the more destabilizing ξ$E_{intra}^{X}$ intra-atomic energies are responsible for the very high RIAE activation energy ξ$E_{total}^{Bu+Et}$.

The inclusion of a strong EW NO_2_ group in 2-nitropropene **17** decreases the ξ$E_{total}^{Et}$ total energies of electrophilic ethylene framework of **TS7** to −23.1 kcal·mol^−1^. Despite the increase in the unfavorable ξ$E_{total}^{Bu}$ total energies of the nucleophilic butadiene framework to 34.0 kcal·mol^−1^, the RIAE activation energy decreases to 10.9 kcal·mol^−1^.

The inclusion of a strong ER OCH_3_ group in 2-methoxypropene **20** and a strong EW NO_2_ group in 2-nitropentadiene **15** causes a more remarkable effect in the RIAE activation energy of **TS10** in the P-DA reaction of **15** with **20**. While the ξ$E_{total}^{Et}$ total energies of the nucleophilic ethylene framework increase to 43.4 kcal·mol^−1^, those of the electrophilic butadiene framework decrease considerably to −36.3 kcal·mol^−1^. These effects reduce the RIAE activation energy of this P-DA reaction to 7.1 kcal·mol^−1^

The molecular structure of the TSs of the three selected IMDA reactions is divided into three frameworks: (a) the ethylene, Et, (b) the butadiene, Bu, and c) the –(CH_2_)_4_- saturated hydrocarbon chain, CH_2_, linking the two unsaturated frameworks (see Table 5). A comparative analysis of the ξ$E_{inter}^{X}$ interatomic, ξ$E_{intra}^{X}$ intra-atomic and ξ$E_{total}^{X}$ total energies of the three DA and the three IMDA reactions shows that both types of reactions experience a similar electronic effect with the substitution at the ethylene and the butadiene frameworks. The RIAE activation energies of the TSs of the IMDA reaction are 3.6 and 6.1 kcal·mol^−1^ higher in energy than those of the intermolecular DA reactions. In general, the unfavorable ξ$E_{intra}^{CH2}$ intra-atomic energies associated with the –(CH_2_)_4_- chain are responsible for these energy differences. The N-IMDA reaction of DTE **5** presents the most unfavorable RIAE activation energy, 24.5 kcal·mol^−1^. The ξ$E_{total}^{X}$ total energies associated with the ethylene and butadiene frameworks are destabilized by ca. 17 kcal·mol^−1^. As in the P-DA reactions, the inclusion of a strong EW NO_2_ group at the ethylene framework of 9-nitro derivative DTE **10**, or a strong EW NO_2_ group at the butadiene framework combined with a strong ER OCH_3_ group at the ethylene framework of 9-methoxy-2-nitro derivative DTE **13**, reduces the RIAE activation energies associated with **TS2** and **TS5** to 17.0 and 11.7 kcal·mol^−1^, respectively. The analysis of the ξ$E_{total}^{X}$ total energies of **TS2** and **TS5** further shows that the stabilization of the electrophilic ethylene at **TS2** or electrophilic butadiene framework at **TS5** is the factor responsible for the acceleration observed in these IMDA reactions. The GEDT taking place at the TSs is also represented.

**Figure 6:**
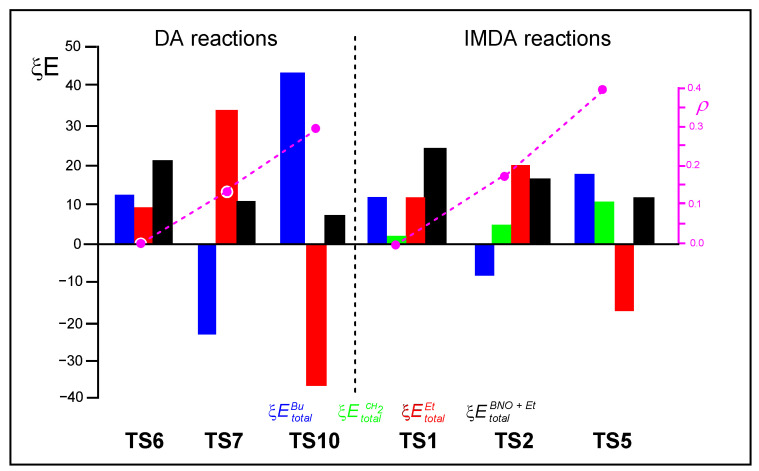
Graphical representation of the sets of the M06-2X/6-311G(d,p) total energies, ξ$E_{total}^{Bu}$, ξ$E_{total}^{CH2}$, ξ$E_{total}^{Et}$, ξ$E_{total}^{Bu+Et}$ and the ξ$E_{total}^{Bu+CH2+Et}$ for the TSs. The ξ$E_{total}^{Bu+Et}$ and ξ$E_{total}^{Bu+CH2+Et}$ energies correspond to the RIAE activation energies of these DA and IMDA reactions, respectively. ξ$E_{total}^{X}$ energies for the Bu, Et and CH2 frameworks are shown in blue, red and green, respectively, while the black bar represents the ξ$E_{total}^{Bu+Et}$ and ξ$E_{total}^{Bu+CH2+Et}$ energies. ξ$E_{total}^{X}$ energies are reported in kcal mol−1. The GEDT values at the TSs, displayed in pink, are expressed as an average number of electrons e.

Finally, Figure 6 shows a graphical representation of the ξ$E_{total}^{X}$ energies of the Bu, Et and CH_2_ frameworks at the six TSs, shown in blue, red and green, respectively, and the ξ$E_{total}^{Bu+Et}$ and ξ$E_{total}^{Bu+CH2+Et}$ energies, shown in black, which correspond to the RIAE activation energies of these DA and IMDA reactions, respectively.

An analysis of the data presented in Figure 6 permits us to obtain some interesting conclusions: (i) in both DA and IMDA reactions, the RIAE activation energies decrease with increasing GEDT. This decrease is more effective for the intermolecular processes; (ii) a comparison of the ξ$E_{total}^{X}\;$ total energies associated with the ethylene and butadiene frameworks in the inter- and intramolecular polar reactions indicates these energies are higher in the intermolecular DA reactions, suggesting more effective nucleophilic/electrophilic interactions in the intermolecular processes, despite the fact that GEDT values are higher in IMDA reactions; (iii) in non-polar cycloaddition reactions, both ethylene and butadiene frameworks are destabilized at the TSs, explaining the high activation energies associated with these non-polar reactions; (iv) in polar reactions, although the nucleophilic frameworks are strongly destabilized compared to the non-polar reactions, the electrophilic frameworks are even more strongly stabilized, reducing the RIAE activation energies of polar reactions; and finally, (v) in IMDA reactions, the ξ$E_{total}^{CH2}\;$ total energies, associated with the strain of the chain, increase as the RIAE activation energies decrease, due to the more advanced character of the IMDA reaction.

## 3. Computational Methods

The M06-2X functional [40], together with the standard 6-311G(d,p) basis set [41], which includes d-type polarization for second-row elements and p-type polarization functions for hydrogen atoms, was used throughout this MEDT study The TSs were identified by the presence of a single imaginary frequency. Optimizations were performed using the Berny algorithm [42,43]. Intrinsic reaction coordinate [44] (IRC) paths were traced to map of the energy profiles linking each TS to the two corresponding minima on the PES.

Solvent effects of THF were considered in the thermodynamic calculations by full optimization of the *in vacuo* structures at the same computational level using the polarizable continuum model (PCM) [45,46] in the framework of the self-consistent reaction field (SCRF) [47,48,49]. Values of M06-2X/6-311G(d,p) enthalpies, entropies and Gibbs free energies in THF were calculated with standard statistical thermodynamics at 66 °C and 1 atm through PCM frequency calculations at the solvent-optimized structures. Except for thermodynamic data, all other results were obtained *in vacuo* for an overall coherence in interpreting the results obtained from different quantum chemical tools.

DFT-based reactivity indices [29,30] were calculated using the equations in [30]. The GEDT [8] values were computed using the equation GEDT(f) = Σq_f_, where q corresponds to the natural charges [27,28] of the atoms belonging to one of the two frameworks (f) at the TS geometries.

The Gaussian 16 suite of programs was used to perform the calculations [50]. Molecular geometries and ELF basin attractors were visualized by using the GaussView program [51]. ELF analyses [26] of the M06-2X/6-311G(d,p) monodeterminantal wavefunctions were done by using the TopMod [52] package with a cubical grid of step size of 0.1 Bohr. The Interacting Quantum Atoms [53] (IQA) analysis for the RIAE study [39] was performed with the AIMAll package [54] using the corresponding M06-2X/6-311G(d,p) monodeterminantal wavefunctions.

## 4. Conclusions

The IMDA reactions of DTEs **5**, **10**, **12** and **13** and MODA **11** have been studied within the MEDT framework at the M06-2X/6-311G(d,p) computational level and compared with the intermolecular DA reactions of the unsaturated compounds **14**–**20** to better understand the behavior of the intramolecular processes.

An ELF topological analysis of the GS electronic structure of DTEs **5**, **10**, **12** and **13** and MODA **11** shows a strong similarity with that of the derivatives **14**–**20**. The inclusion of EW or ER groups in these compounds does not modify markedly the electronic structure of non-substituted compounds.

The analysis of the electrophilicity ω and nucleophilicity *N* indices of DTEs **5**, **10**, **12** and **13** and MODA **11** indicates that they are closely related to those of the butadiene and ethylenes derivatives **14**–**20**. On the other hand, the analysis of the local reactivity difference R_k_ indices permits the study of the regioselectivity in intramolecular polar processes helping to identify the most nucleophilic and electrophilic centers of these complex molecules.

The analysis of the relative energies of the stationary points involved in the most favorable reaction path associated with the IMDA reactions of DTEs **5**, **10**, **12** and **13** and MODA **11** leads to two significant conclusions: (i) the five IMDA reactions are strongly exothermic in the range −43.8 (**5**) to −52.2 (**11**) kcal·mol^−1^, while the relative energies of the TSs range from 23.9 for the N-IMDA reaction of DTE **5** via **TS1** kcal·mol^−1^ to −0.8 kcal·mol^−1^ for the I-IMDA reaction of MODA **11** via **TS3**. The trend in these relative energies closely resembles that of DA reactions; and (ii) the REDF P-IMDA reactions of DTEs **12** and **13** demand double substitution at the diene and ethylene frameworks to facilitate the polar reactions.

Analysis of the geometries, GEDT values, and electronic structure of the five TSs involved in the IMDA reactions shows a strong similarity to those found in the intermolecular DA reactions, indicating that the substituents have similar electronic effects in both types of DA reactions.

A comparative RIAE analysis of the intermolecular and intramolecular DA reactions allows for the establishment of the effects of the EW and ER groups in these polar reactions. The non-polar reactions are highly disfavored due to the unfavorable ξ$E_{intra}^{X}$ intra-atomic energies associated with the butadiene and ethylene frameworks demanded to reach the TSs. The polar character of the TS enhances the feasibility of these DA reactions. As the butadiene framework of DTE **5** is nucleophilically activated, the ethylene framework must be electrophilically activated by the presence of an EW NO_2_ group. The RIAE analysis shows that although the nucleophilic frameworks are destabilized due to the loss of electron density, the electrophilic frameworks are further stabilized due to the gain of electron density, thereby reducing the RIAE activation energies.

The present MEDT study shows the strong electronic similarity between the intramolecular and intermolecular DA reactions. The non-substituted compounds demand a high energy cost to reach the non-polar TSs. To favor the bonding changes in these reactions, at least one of the two interacting frameworks should be electrophilically activated by the presence of an EW group, such as NO_2_, to increase the GEDT of the reaction. The same trend is observed in both types of DA reactions. The main difference between them lies in the unfavorable activation entropy associated with intermolecular DA reactions. Thus, the lower activation entropy associated with the intramolecular processes makes them approximately 10^4^ times faster than intermolecular ones, despite the fact that the activation enthalpies associated with the latter are lower.

## Figures and Tables

**Table 1:** B3LYP/6-31G(d) electronic chemical potential μ, chemical hardness η, and electrophilicity ω and nucleophilicity *N* indices, calculated at the B3LYP/6-31G(d) for DTEs **5**, **10**, **12** and **13** and MODA **11**, expressed in eV. The reactivity indices of the MODA cation **11** are also computed in DMSO.

	C2	C(N)5	μ	η	ω	*N*
**11**	H	H^+^	−7.80	0.86	35.28	0.89
**11** (DMSO)			−4.46	3.31	3.00	2.64
**10**	H	NO_2_	−4.23	3.60	2.49	3.09
**12**	NO_2_	OCH_3_	−4.28	3.76	2.43	2.97
**13**	NO_2_	CH_3_	−4.48	4.21	2.38	2.54
**5**	H	H	−3.19	5.47	0.93	3.19

**Table 2:** Electrophilic $P_{k}^{+}\;$ and nucleophilic $P_{k}^{-}$ Parr functions, and reactivity difference R_k_ indices of DTEs **10**, **12** and **13** and MODA **11**.

	$P_{k}^{+}$	$P_{k}^{-}$	R_k_	$P_{k}^{+}$	$P_{k}^{-}$	R_k_
k		**10**			**11**	
**1**	0.10	0.38	−0.94	0.00	0.47	−1.23
**2**	0.02	0.00	0.00	0.00	0.07	−0.19
**3**	0.01	0.14	−0.40	0.00	0.10	−0.25
**4**	0.09	0.29	−0.68	0.00	0.40	−1.06
**5**	−0.02	0.02	−0.12	0.25	0.00	0.74
**6**	0.31	0.04	0.45	0.85	0.00	2.56
k		**12**			**13**	
**1**	0.47	0.20	0.62	0.46	0.16	0.63
**2**	−0.02	−0.02	0.00	−0.01	−0.01	0.00
**3**	0.00	0.14	−0.36	0.00	0.11	−0.33
**4**	0.08	0.17	−0.24	0.11	0.14	−0.14
**5**	0.00	0.12	−0.30	0.00	0.04	−0.11
**6**	0.00	0.28	−0.70	0.00	0.33	−0.97

**Table 3:** M06-2X/6-311G(d,p) gas phase relative energies, in kcal·mol^−1^, of the stationary points involved in the IMDA reactions of DTEs **5**, **10**, **12** and **13** and MODA **11**.

NEDF		FEDF		REDF	
**5**	0.0	**10**	0.0	**12**	0.0
**TS1**	23.9	**TS2**	17.1	**TS4**	16.5
**21**	−43.8	**22**	−45.4	**24**	−47.7
		**11**	0.0	**13**	0.0
		**TS3**	−0.8	**TS5**	12.0
		**23**	−52.2	**25**	−49.9

**Table 4:** M06-2X/6-311G(d,p) activation Gibbs free energies, ΔG^‡^ in kcal·mol^−1^, calculated in tetrahydrofuran at 66 °C, along with the RRRCs, k_r_, for the DA reactions of compounds **14**–**20**, and for the IMDA reactions of DTEs **5**, **10**, **12** and **13** and MODA **11**. $k_{r}^{I}$ represents the RRRCs relative to **TS7**, while $k_{r}^{2}$ represents the RRRCs of the IMDA reactions relative to the corresponding DA reactions.

		ΔG^‡^	$k_{r}^{I}$	$k_{r}^{2}$
**14 + 16**	**TS6**	38.9	1.5 × 10^−7^	
**14 + 17**	**TS7**	28.3	1.0	
**14 + 18**	**TS8**	18.9	1.1 × 10^6^	
**15 + 19**	**TS9**	30.2	6.0 × 10^−2^	
**15 + 20**	**TS10**	21.7	1.8 × 10^4^	
**5**	**TS1**	30.4	5.6 × 10^−5^	3.0 × 10^5^
**10**	**TS2**	23.8	1.0	7.9 × 10^2^
**11**	**TS3**	12.8	1.2 × 10^7^	8.5 × 10^3^
**12**	**TS4**	21.9	1.7 × 10^1^	2.2 × 10^5^
**13**	**TS5**	16.2	7.9 × 10^4^	3.5 × 10^3^

**Table 5:** M06-2X/6-311G(d,p) gas-phase total energies, ξ$E_{total}^{X}$, intra-atomic energies, ξ$E_{intra}^{X}$, and interatomic energies, ξ$E_{inter}^{X}$ calculated using M06-2X/6-311G(d,p) level for the ethylene, Et, butadiene, Bu, and the –(CH_2_)_4_– chain, CH_2_, frameworks at the selected TSs relative to their GSs. The sum of the ξ$E_{total}^{X}$ energies of both frameworks, denoted as ξ$E_{total}^{Bu+CH2+Et}$, represents the RIAE activation energies.

TS	*f(x)*	$\xi E_{intra}^{X}$	$\xi E_{inter}^{X}$	$\xi E_{total}^{X}$	$\xi E_{total}^{Bu+CH2+Et}$
**TS6**	Et	36.2	−23.8	12.4	20.9
	Bu	38.3	−29.8	8.5	
**TS7**	Et	31.8	−54.9	−23.1	10.9
	Bu	52.8	−18.8	34.0	
**TS10**	Et	95.9	−52.4	43.4	7.1
	Bu	43.5	−79.7	−36.3	
**TS1**	Et	29.3	−17.6	11.7	24.5
	CH_2_	5.2	−3.4	1.8	
	Bu	36.2	−25.2	11.0	
**TS2**	Et	36.7	−44.7	−8.0	17.0
	CH_2_	5.8	−1.4	4.4	
	Bu	41.3	−20.6	20.6	
**TS5**	Et	81.1	−63.2	17.8	11.7
	CH_2_	3.9	6.9	10.8	
	Bu	42.0	−59.0	−17.0	

## Data Availability

The data that support the findings of this study are available from the corresponding author upon reasonable request.

## Supplementary Materials

The following supporting information can be downloaded at: https://www.mdpi.com/article/10.3390/molecules30092052/s1, ELF study of the electronic structure of compounds **14**–**18**, **20**. Analysis of the chemical properties of compounds **14**–**20**. Study of the DA reactions of compounds **14**–**20**. Theoretical background of the Relative Interacting Atomic Energy (RIAE) Analysis. References. Tables with the M06-2X/6-311G(d,p) total and relative energies, enthalpies, entropies, and Gibbs free energies, for the stationary points involved in the IMDA reactions of DTEs **5**, **10**, **12** and **13** and MODA **11**, and in the DA reactions of compounds **14**–**20**. M06-2X/6-311G(d,p) gas phase computed total energies and Cartesian coordinates of the stationary points involved in the IMDA reactions of DTEs **5**, **10**, **12** and **13** and MODA **11**, and in the DA reactions of compounds **14**–**20**.
