# Supplementary material for: Intramolecular Versus Intermolecular Diels–Alder Reactions: Insights from Molecular Electron Density Theory
Source: Molecules. 2025 May 5;30(9):2052. doi: 10.3390/molecules30092052 (PMC12073351; doi:10.3390/molecules30092052)
Supplement: Supplementary file 1 [file molecules-30-02052-s001.zip › molecules-3617132-supplementary.pdf]

Supplementary Material

Index

S2 ELF study of the electronic structure of compounds **14** – **18**, **20**.

S4 Analysis of the chemical properties of compounds **14** – **20**.

S5 Study of the DA reactions of compounds **14** – **20**.

S8 Theoretical background of the Relative Interacting Atomic Energy (RIAE) Analysis

S10 References

S12 Table with the M06-2X/6-311G(d,p) gas phase total energies of the stationary points involved in the IMDA reactions of DTEs **5**, **10**, **12** and **13** and MODA **11**.

S12 Table with the M06-2X/6-311G(d,p) total and relative energies, enthalpies, entropies, and Gibbs free energies, computed in tetrahydrofuran at 66 ºC, for the stationary points involved in the IMDA reactions of DTEs **5**, **10**, **12** and **13** and MODA **11**.

S13 Table with the M06-2X/6-311G(d,p) total and relative energies, enthalpies, entropies, and Gibbs free energies, computed in tetrahydrofuran at 66 ºC, for the stationary points involved in the DA reactions of compounds **14** – **20**.

S14 M06-2X/6-311G(d,p) gas phase computed total energies and Cartesian coordinates of the stationary points involved in the IMDA reactions of DTEs **5**, **10**, **12** and **13** and MODA **11**.

S24 M06-2X/6-311G(d,p) gas phase computed total energies and Cartesian coordinates of the stationary points involved in the DA reactions of compounds **14** – **20**.

*1. ELF study of the electronic structure of compounds **14 – 18, 20**.*

The attractor positions of the electron localization function[1] (ELF) basins, the populations of the most relevant valence basins, and the natural atomic charges of compounds **14** – **18**, **20** involved in the DA reactions are shown in Figure S1.

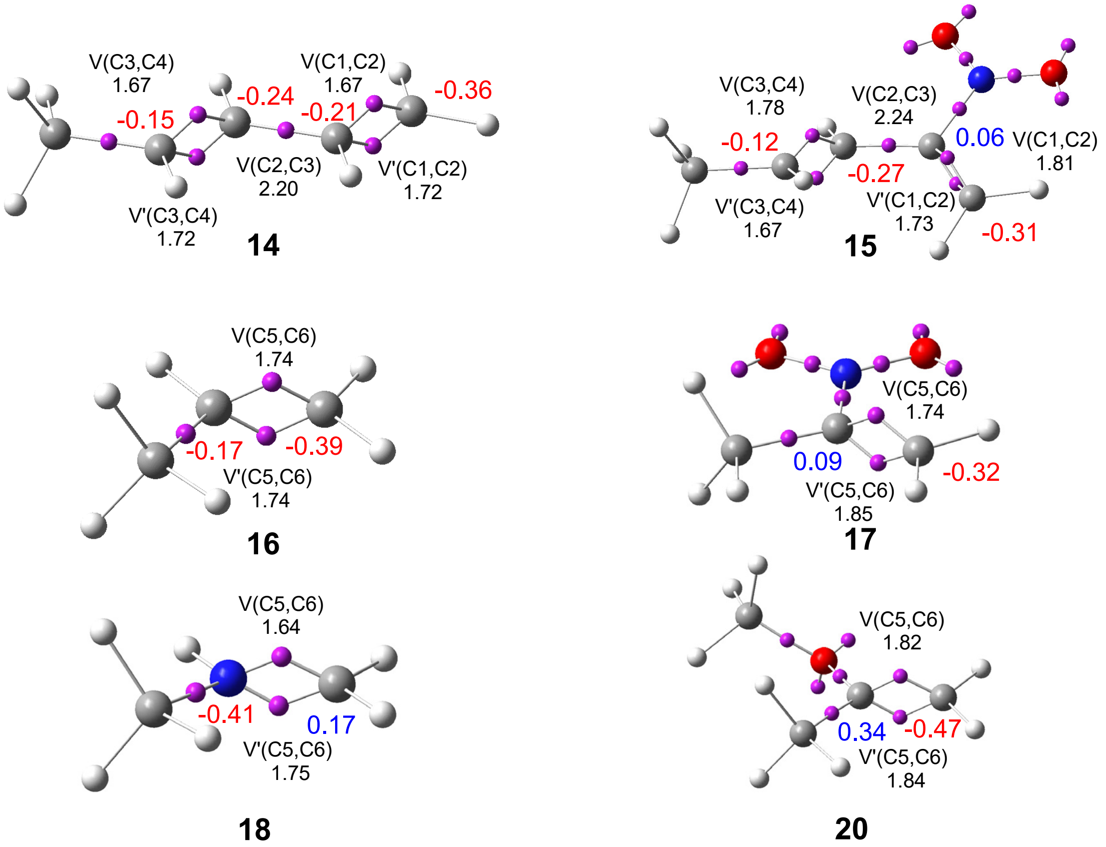

Figure S1. ELF basin attractor positions and the populations of the most relevant valence basins of compounds **6 - 11,** calculated at the M06-2X/6-311G(d,p) level in the gas phase. Natural atomic charges are reported as the average number of electrons, e. Negative charges are highlighted in red, positive charges in blue, and negligible charges in green.

ELF topological analysis of 1,3-pentadienes **14** and **15** shows the presence of two disynaptic basins, V(C1,C2) and V'(C1,C2), integrating a total of 3.39 and 3.54 e, respectively, associated with a depopulated C1−C2 double bond. Additionally, one V(C2,C3) disynaptic basin integrating ca. 2.2 e, corresponding to a populated C2–C3 single bond. Furthermore, two monosynaptic basins, V(C3,C4) and V'(C3,C4), integrating a total of 3.39 and 3.45 e, respectively, associated with a depopulated C3–C4 double bond.

ELF of ethylenes **16**, **17** and **20** shows the presence of two disynaptic basins, V(C5,C6) and V'(C5,C6), integrating a total of 3.58, 3.53 and 3.66 e, respectively, associated with a depopulated C5−C6 double bond. Finally, ELF of the iminium cation **18** shows the presence of two disynaptic basins, V(N5,C6) and V'(N5,C6), integrating a total of 3.39 e, associated with a depopulated N5−C6 double bond.

The natural atomic charges[2,3] of the most relevant centers of compounds **14** – **18**, **20** are presented in Figure S1. In general, all carbon belonging to the unsaturated framework of pentadienes **14** and **15** are negatively charged between −0.12 and −0.36 e. The negative charge on the carbon atoms depends on the number of hydrogen atoms attached to them; this behavior is caused by the more electronegative character of the carbon atom than the hydrogen atom.

The presence of the NO_2 or the OCH_3 groups in the pentadiene **15** and the propene derivatives **17** and **20** induces significant changes in the natural atomic charges of the carbon atom to which they are attached. Specifically, when NO_2 or OCH_3 groups are located at the C2 or C5 positions in these compounds, the corresponding carbon atoms become positively charged. (see Figure S1). This behavior is primarily a consequence of the higher electronegativity of the N nitrogen or O oxygen atoms compared to C carbon one, rather than the electron-withdrawing (EW) or electron-releasing (ER) nature of the substituent groups. Interestingly, at the iminium cation **18** formed by protonation of the N5 nitrogen in the corresponding imine is negatively charged by −0.41 e.

*2. Analysis of the chemical properties of compounds **14 – 20***

Table S1 summarizes the B3LYP/6-31G(d) DFT-based reactivity indices[4,5] including electronic chemical potential μ, chemical hardness η, as well as the electrophilicity ω and nucleophilicity *N* indices of compounds **14** – **20**. The reactivity index of iminium cation **17** was computed in DMSO.[6]

Table S1. Electronic chemical potential μ, chemical hardness η, and electrophilicity ω and nucleophilicity *N* indices, calculated at the B3LYP/6-31G(d) for the compounds **14** – **20** involved in the intermolecular Diels-Alder reactions, expressed in eV. The reactivity indices of the iminium cation **18** computed in DMSO are given in parenthesis.

	μ	η	ω	*N*
iminium cation **18**	-11.83	7.80	8.97	-6.61
iminium cation (DMSO) **18**	-6.80	7.94	2.91	-2.02
2-nitro-pentadiene **15**	-4.66	4.47	2.43	2.23
2-nitro-propene **17**	-5.16	5.48	2.43	1.22
1,3-butadiene **27**	-3.42	5.62	1.04	2.89
1,3-pentadiene **14**	-3.17	5.51	0.91	3.20
ethylene **28**	-3.37	7.77	0.73	1.87
propene **16**	-3.01	7.57	0.60	2.32
isobutene **19**	-2.83	7.37	0.55	2.60
2-methoxy-propene **20**	-2.40	6.97	0.41	3.24

The electrophilicity ω and nucleophilicity *N* indices of 1,3-pentadiene **14** are 0.91 and 3.20eV, respectively, classifying it as a moderate electrophile and a strong nucleophile within the electrophilicity and nucleophilicity scales. On the other hand, the electrophilicity ω and nucleophilicity *N* indices of propene **16** are 0.60 and 2.30 eV, respectively, classifying it as a moderate electrophile and a moderate nucleophile. Consequently, only 1,3-pentadiene **14** could participate as a strong nucleophile in polar DA reactions.

The presence of a strong EW NO_2 group in compounds **15** and **17** notably increases the electrophilicity ω index of the corresponding nitro derivatives to 2.43 eV, classifying both nitro derivatives as strong electrophiles. On the other hand, the presence of a strong ER OCH_3 group in 2-methoxy-propene **20** increases its nucleophilicity *N* index to 3.42 eV, classifying it as a strong nucleophile. Finally, the high electrophilicity ω index of the iminium cation **18** in DMSO, 2.91 eV, classifies this species as a strong electrophile.[6]

2. Study of the DA reactions of compounds ***14** – **20***

The potential energy surfaces (PESs) of the most favorable reaction paths associated with the DA reactions of compounds **14** – **20** were studied (see Scheme S1). These reaction paths correspond to the *endo* approach modes of the substituent present on the ethylene with respect to the butadiene system. Along each one of these DA reactions, one reagent, one TS, and one cycloadduct were located and characterized. This confirms that these DA reactions take place through a one-step mechanism. The M06-2X/6-311G(d,p) gas phase total and relative energies of the stationary points involved in these DA reactions of compounds **14** – **20** are given in Table S2.

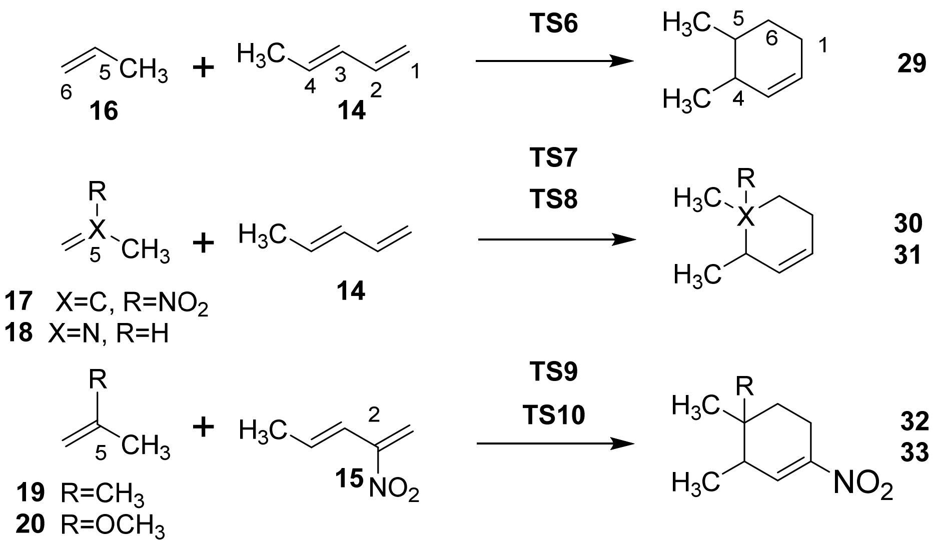

Scheme S2. DA reactions of compounds **14** – **20**.

Some appealing conclusions can be obtained from the relative energies presented in Table S2: i) all five DA reactions are highly exothermic, with reaction energies ranging from –42.8 (**32**) τo –54.8 (**31**) kcal·mol^{-1}. Consequently, these DA reactions can be considered irreversible and kinetically controlled; ii) the relative energies of the TSs ranging from 21.0 kcal·mol^{-1} for the N-DA reaction of **14** with **16** to –9.9 kcal·mol^{-1} for the I-DA reaction of **14** with the iminium cation **18**; iii) these relative energies indicate that substitutions on the unsaturated systems influence in the kinetics more significantly than the thermodynamic of these DA reactions; iv) **TS3** is located –9.9 kcal·mol^{-1} below the separated reagent in the gas phase;[7] however, when solvent effects of THF are considered, the activation energy becomes positive by 7.5 kcal·mol^{-1} due to a larger solvation of the iminium cation **18** compared to **TS3** (see Table S4); v) in the REDF P-DA reactions, double substitution is necessary to increase the nucleophilic character of the propene **16** and to change the nucleophilic character of pentadiene **14** into an

electrophilic one; and finally, vi) the doble substitution in the REDF P-DA reaction of **15** with **20**, ΔE(**TS10**) = 7.2 kcal·mol^{-1}, is more effective than mono substitution in the FEDF P-DA reaction of **14** with **17**, ΔE(**TS7**) = 11.0 kcal·mol^{-1}.

Table S2. M06-2X/6-311G(d,p) gas phase total energies, E in a.u., and relative energies, ΔE, in kcal·mol^{-1}, for the stationary points involved in the DA reactions of compounds **14** – **20**.

Type	NEDF			FEDF			REDF	
	E	ΔE		E	ΔE		E	ΔE
**14**	-195.257237					**15**	-399.737440	
**16**	-117.870494		**17**	-322.357059		**19**	-157.179386	
**TS6**	-313.094313	21.0	**TS7**	-517.596762	11.0	**TS9**	-556.894263	14.2
**29**	-313.199448	-45.0	**30**	-517.689618	-47.3	**32**	-556.985026	-42.8
			**18**	-134.261883		**20**	-232.380259	
			**TS8**	-329.534895	-9.9	**TS10**	-632.106279	7.2
			**31**	-329.606481	-54.8	**33**	-632.198026	-50.4

Figure S2 shows the gas phase geometries of the TSs involved in the DA reactions of compounds **14** – **20**. While the C1–C6 distances are in the range from 2.15 to 1.91 Å, the C1–C6 distances are in the range from 2.32 to 3.38 Å. These values indicate that all TSs correspond with asynchronous C–C single bond formation process, with increasing asynchronicity as the polar character of the DA reaction (see later). In P-DA and I-DA reactions, the TSs are characterized by the most favorable two-center interaction between the most nucleophilic and electrocyclic centers of the corresponding reactants. These centers correspond to the β position of the substituted C–C double bond. On the other hand, the most favorable **TS8** and **TS10** are slightly more advanced and more asynchronous than the others. The inclusion of THF solvent effects does not induce remarkable changes in the TS geometries (see Figure S2). In THF, the TSs are slightly more advanced and more asynchronous compared to the gas-phase TSs.

Analysis of the global electron density transfer[8] (GEDT) at the TSs involved in these DA reactions enables the quantification of the polar character of these cycloaddition reactions.[9] The gas phase GEDT values calculated for all TSs are presented in Figure S2. While the value of the GEDT at **TS6**, 0.01 e, indicates the non-polar character of this DA reaction, classified as NEDF, **TS8**, associated with the I-DA reaction, presents the maximum value, 0.43 e. The positive values of the GEDT found at **TS7** and **TS8** classify them as FEDF. Finally, the negative values of the GEDT at **TS9** and **TS10** classify them

as REDF. **TS10**, with a GEDT = –0.41 e, correspond with the most asynchronous TS of the three polar ones. The inclusion of solvent effects in THF slightly increases the GEDT at the polar TS, enhancing the electron density transfer process (see Figure S2).

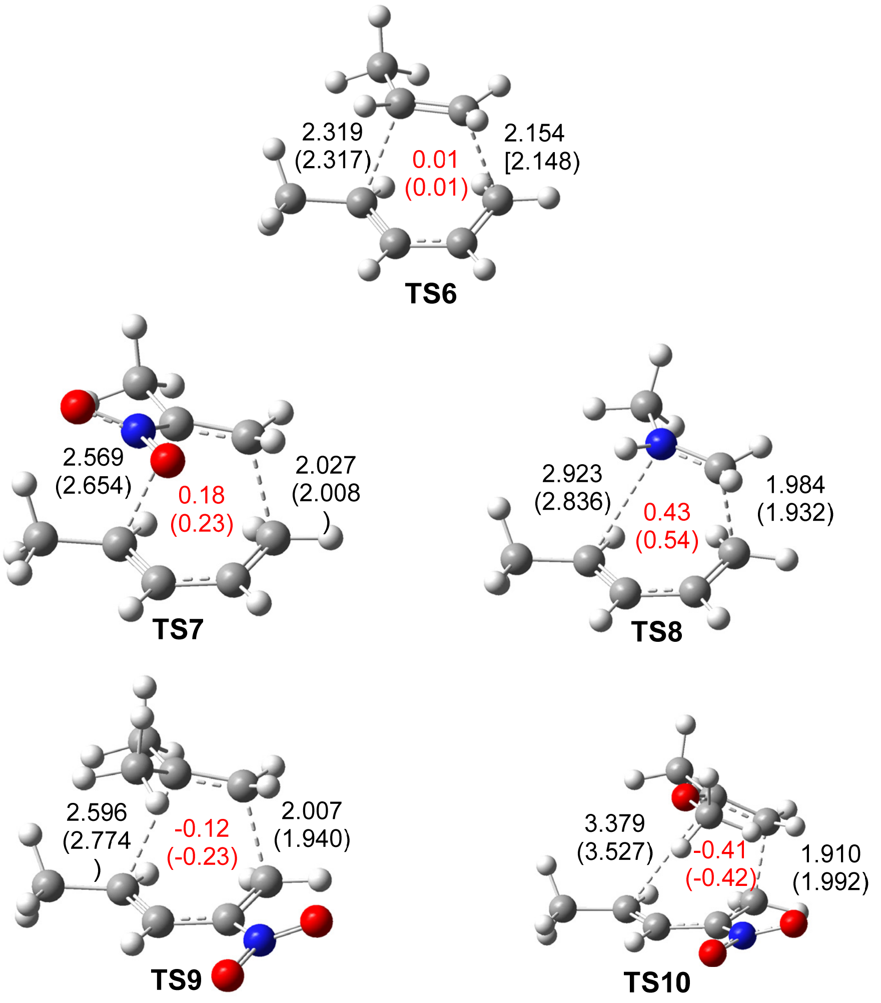

Figure S2. M06-2X/6-311G(d,p) gas phase optimized geometries of **TS6** – **TS10** associated with the DA reactions of compounds **14** – **20**. The GEDT values, in red, are given as the average number of electrons, e. Bond lengths are given in Angstroms, with values in THF given in parenthesis.

4. Theoretical background of the Relative Interacting Atomic Energy (RIAE) Analysis.

The Interacting Quantum Atoms[10] (IQA), based on the Quantum Theory of Atoms in Molecules[11,12] (QTAIM), divides the total energy E_{total}^{IQA} into two main energy contributions: the intra-atomic energies E_{intra}^{A} and the interatomic energies E_{inter}^{AB} (see Equation S1). The E_{inter}^{AB} energies are, in turn, divided into four additional electrostatic terms: the nuclei-electron interactions V_{ne}^{AB} and V_{en}^{AB}, the electron-electron interactions V_{ee}^{AB} , and the nuclei-nuclei interactions V_{nn}^{AB}(see Equation S3).

$$E_{total}^{IQA} = \sum E_{intra}^{A} + \sum E_{inter}^{AB} \tag{S1}$$

$$E_{intra}^{A} = T(A) + V_{ne}^{A} + V_{ee}^{A} \tag{S2}$$

$$E_{inter}^{AB} = ½V_{ne}^{AB} + ½V_{en}^{AB} + ½V_{ee}^{AB} + V_{nn}^{AB} \tag{S3}$$

Thanks to the additivity of topological atoms,[13] an Interacting Quantum Fragments, IQF, approach has been introduced,[14] which allows the grouping of the IQA energy into terms of convenient fragments of the system. This enables a more chemically meaningful analysis of the interactions that take place between the atoms forming molecular groups. In this sense, for RIAE analysis of P-DA reactions, the atoms belonging to the TSs are regrouped into two interacting frameworks f(X) corresponding to the butadiene (Bu) and ethylene (Et) reagents. On the other hand, the molecular structure of the TSs of the three selected intramolecular Diels-Alder IMDA reactions is divided into three frameworks: a) the butadiene (Bu), b) the ethylene (Et) and c) the –$(CH_2)_4$- saturated hydrocarbon chain (CH_2) linking the two unsaturated frameworks.

By default, the sum of all IQA atomic energies belonging to a given framework f(X) (where X represents either the pentadiene or ethylene frameworks) at the TSs, and those of the separated reagents at the ground states (GSs), are computed.[15] The RIAEs, which include the relative total energy, ξE_{total}^{X}, intra-atomic energy, ξE_{intra}^{X} and interatomic energy, ξE_{inter}^{X} , are obtained using Equations S4-S6. The symbol ξ denotes the IQA energy differences between the GS and TS states of the two-interacting frameworks f(X); i.e. f(Bu) and f(Et) in DA reactions, and f(Bu), f(Et) and f(CH_2) in IMDA reactions.

$$\xi E_{total}^{X} = \xi E_{intra}^{X} + \xi E_{inter}^{X} \tag{S4}$$

$$\xi E_{intra}^{X} = \sum E_{intra}^{X(TS)} - \sum E_{intra}^{X(GS)} \tag{S5}$$

$$\xi E_{inter}^{X} = \sum E_{inter}^{X(TS)} - \sum E_{inter}^{X(GS)} \tag{S6}$$

The RIAE analysis quantifies the degree of destabilization or stabilization of the interacting frameworks f(X) as they transition from their GS to the TS. A positive relative

energy indicates destabilization, whereas a negative relative energy signifies stabilization. The sum of the ξE_{total}^{X} energies of the two interacting frameworks, ξE_{total}^{Bu+Et} or $\xi E_{total}^{Bu+Et+CH2}$ represents the RIAE activation energy of DA and IMDA reactions as determined by the present energy decomposition analysis.[15-17]

Refecences

1. Becke, A.D.; Edgecombe, K.E. A simple measure of electron localization n atomic and molecular-systems. *J. Chem. Phys.* **1990**, *92*, 5397–5403.
2. Reed, A. E.; Weinstock, R. B.; Weinhold, F. Natural population analysis, *J. Chem. Phys.* **1985**, *83*, 735–746.
3. E. Reed, L. A. Curtiss and F. Weinhold, Intermolecular interactions from a natural bond orbital, donor-acceptor viewpoint. *Chem. Rev.* **1988**, *88*, 899–926.
4. Parr, R.G.; Yang, W. *Density functional theory of atoms and molecules*, Oxford University Press, New York, 1989.
5. Domingo, L.R.; Ríos-Gutiérrez, M.; Pérez, P. Applications of the conceptual density functional indices to organic chemistry reactivity. *Molecules* **2016**, *21*, 748.
6. Domingo, L.R.; Ríos-Gutiérrez, M.; Pérez, P. Electrophilicity ω and Nucleophilicity *N* Scales for Cationic and Anionic Species. *Sci. Rad.* **2025**, *4*, 1-17.
7. Domingo, L.R.; Ríos-Gutiérrez, Aurell, M.J. Unveiling the Ionic Diels-Alder Reactions within the Molecular Electron Density Theory. Molecules **2021**, *26*, 3638.
8. Domingo, L.R. A new C–C bond formation model based on the quantum chemical topology of electron density. *RSC Adv*. **2014**, *4*, 32415–32428.
9. Domingo, L.R.; Sáez, J.A. Understanding the Mechanism of the Polar Diels-Alder Reaction. *Org. Biomol. Chem.* **2009**, *7*, 3576–3583.
10. Blanco, M.A.; Martín Pendás, A.; Francisco, E. Interacting Quantum Atoms: A Correlated Energy Decomposition Scheme Based on the Quantum Theory of Atoms in Molecules. *J. Chem. Theory Comput.* **2005**, *1*, 1096–1109.
11. Bader, R.F.W.: Tang, Y.H.; Tal, Y.; Biegler-König, F.W. Properties of atoms and bonds in hydrocarbon molecules. *J. Am. Chem. Soc*. **1982**, *104*, 946–952.
12. Bader, R.F.W. Atoms in Molecules: A Quantum Theory. Oxford University Press, Oxford, New York, 1994.
13. Wilson, A.L.; Popelier, P.L.A. Exponential Relationships Capturing Atomistic Short-Range Repulsion from the Interacting Quantum Atoms (IQA) Method. *J. Phys. Chem. A*, **2016**, *120*, 9647–9659.
14. Triestram, L.; Falcioni, F.; Popelier, P.L.A. Interacting Quantum Atoms and Multipolar Electrostatic Study of XH···π Interactions. *ACS Omega* **2023,** *8*, 34844–34851.

15. Domingo, L.R.: Ríos-Gutiérrez, M.; Pérez, P.; Understanding the Electronic Effects of Lewis Acid Catalysts in Accelerating Polar Diels-Alder Reactions. *J. Org. Chem.* **2024**. 89, 12349-12359.
16. Domingo, L.R.; Pérez, P.; Ríos-Gutiérrez, M.; Aurell, M.J. A Molecular Electron Density Theory Study of Hydrogen Bond Catalysed Polar Diels–Alder Reactions of α,β-unsaturated Carbonyl Compounds, *Tetrahedron Chem.* **2024**, *10*,100064.
17. Domingo, L.R.; Ríos-Gutiérrez, Revealing the Decisive Role of Global Electron Density Transfer in the Reaction Rate of Polar Organic Reactions within Molecular Electron Density Theory, *Molecules* **2024**, *29*, 1.

Table S3. M06-2X/6-311G(d,p) gas phase total energies, in a.u, of the stationary points involved in the IMDA reactions of DTEs **5**, **10**, **12** and **13** and MODA **11**.

	NEDF		FEDF		REDF
**5**	-390.543538	**10**	-595.030867	**12**	-634.330734
**TS1**	-390.505402	**TS2**	-595.003599	**TS4**	-634.304457
**21**	-390.613264	**22**	-595.103229	**24**	-634.406715
		**11**	-406.944387	**13**	-709.532926
		**TS3**	-406.945696	**TS5**	-709.513856
		**23**	-407.027600	**25**	-709.612524

Table S4. M06-2X/6-311G(d,p) total energies, E in a.u., enthalpies, H in a.u., entropies, S in cal·mol^{-1}·K^{-1}, and Gibbs free energies, G in a.u., along with relative energies, ΔE in kcal·mol^{-1}, enthalpies, ΔH in kcal·mol^{-1}, entropies, ΔS in cal·mol^{-1}·K^{-1}, and Gibbs free energies, ΔG in kcal·mol^{-1}, computed in tetrahydrofuran at 66 ºC, for the stationary points involved in the IMDA reactions of DTEs **5**, **10**, **12** and **13** and MODA **11**.

	E	ΔE	H	ΔH	S	ΔS	G	ΔG
**5**	-390.298115		-390.297041		117.3		-390.360392	
**TS1**	-390.259895	24.0	-390.258821	24.0	98.5	-18.8	-390.312008	30.4
**21**	-390.362157	-40.2	-390.361084	-40.2	94.6	-22.7	-390.412171	-32.5
**10**	-594.783092		-594.783092		132.3		-594.853508	
**TS2**	-594.756844	16.5	-594.755771	17.1	110.7	-21.7	-594.815544	23.8
**22**	-594.850352	-42.2	-594.849279	-41.5	107.9	-24.5	-594.907540	-33.9
**11**	-407.022753		-406.769494		115.46		-406.831869	
**TS3**	-407.009508	8.3	-406.757562	7.5	99.726	-15.7	-406.811437	12.8
**23**	-407.092746	-43.9	-406.835345	-41.3	94.553	-20.9	-406.886426	-34.2
**12**	-634.052778		-634.051704		140.7		-634.127719	
**TS3**	-634.028714	15.1	-634.027641	15.1	120.7	-20.0	-634.092829	21.9
**24**	-634.124347	-44.9	-634.123273	-44.9	115.1	-25.6	-634.185474	-36.2
**13**	-709.250639		-709.249565		146.3		-709.328602	
**TS4**	-709.235360	9.6	-709.234286	9.6	126.7	-19.6	-709.302719	16.2
**25**	-709.325414	-46.9	-709.324341	-46.9	120.9	-25.4	-709.389633	-38.3

Table S5. M06-2X/6-311G(d,p) total energies, E in a.u., enthalpies, H in a.u., entropies, S in cal·mol^{-1}·K^{-1}, and Gibbs free energies, G in a.u., along with relative energies, ΔE in kcal·mol^{-1}, enthalpies, ΔH in kcal·mol^{-1}, entropies, ΔS in cal·mol^{-1}·K^{-1}, and Gibbs free energies, ΔG in kcal·mol^{-1}, computed in tetrahydrofuran at 66 ºC, for the stationary points involved in the DA reactions of compounds **14** – **20**.

	E	ΔE	H	ΔH	S	ΔS	G	ΔG
**16**	-117.786713		-117.785640		65.3		-117.820893	
**14**	-195.138019		-195.136945		78.7		-195.179458	
**TS6**	-312.887469	23.4	-312.886396	22.7	96.3	-47.6	-312.938429	38.9
**29**	-312.986589	-38.8	-312.985515	-39.5	90.8	-53.2	-313.034561	-21.5
**17**	-322.271544		-322.270471		80.8		-322.314105	
**TS7**	-517.390542	11.9	-517.389469	11.3	109.3	-50.2	-517.448498	28.3
**30**	-517.475702	-41.5	-517.474628	-42.2	104.1	-55.4	-517.530844	-23.4
**18**	-406.770568		-406.769494		115.5		-406.831869	
**TS8**	-406.758635	7.5	-406.757562	7.5	99.7	-15.7	-406.811437	12.8
**31**	-406.836419	-41.3	-406.835345	-41.3	94.6	-20.9	-406.886426	-34.2
**15**	-399.615964		-399.614890		95.2		-399.666308	
**19**	-157.065879		-157.064806		73.6		-157.104541	
**TS9**	-556.658852	14.4	-556.657778	13.8	120.4	-48.4	-556.722797	30.2
**32**	-556.741463	-37.4	-556.740389	-38.1	112.9	-55.8	-556.801373	-19.2
**20**	-232.262614		-232.261541		79.2		-232.304330	
**TS10**	-631.868769	6.2	-631.867695	5.5	127.1	-47.3	-631.936365	21.5
**33**	-631.949838	-44.7	-631.948765	-45.4	119.2	-55.2	-632.013133	-26.7

M06-2X/6-311G(d,p) gas phase computed total energies and Cartesian coordinates of the stationary points involved in the IMDA reactions of DTEs **5**, **10**, **12** and **13** and MODA **11**.

5

E(RM062X) = -390.543537638 A.U.

1	6	-0.403849	-0.758474	0.231804
2	6	-0.190456	0.096991	1.233564
3	6	0.997391	0.077442	2.080714
4	6	1.211447	0.927778	3.083907
5	6	-3.262315	-0.302911	-5.430015
6	1	-3.778164	-1.260975	-5.471774
7	1	-0.934104	0.861778	1.451893
8	1	1.736468	-0.688490	1.857274
9	1	0.489556	1.700505	3.327321
10	1	0.350990	-1.516641	0.023576
11	6	-1.602130	-0.749353	-0.666699
12	6	-2.070361	-0.238057	-4.521157
13	6	-2.440676	-0.512170	-3.059338
14	6	-1.229626	-0.485765	-2.130845
15	1	-2.113987	-1.717550	-0.604620
16	1	-0.500433	-1.235292	-2.460118
17	1	-2.313241	0.009414	-0.325458
18	1	-0.729466	0.486047	-2.201410
19	1	-1.598124	0.745900	-4.603998
20	1	-1.326745	-0.978283	-4.840270
21	1	-3.174285	0.232668	-2.732487
22	1	-2.935136	-1.488037	-2.986651
23	1	-3.239247	1.683137	-6.126544
24	1	-4.602617	0.613066	-6.775224
25	1	2.107617	0.879081	3.689270
26	6	-3.726524	0.713169	-6.145797

10

E(RM062X) = -595.030866583 A.U.

1	6	-0.477720	-0.746363	0.321161
2	6	-0.146842	0.146259	1.256299
3	6	1.033838	0.034590	2.106330
4	6	1.364017	0.923533	3.042225
5	6	-3.250079	-0.275551	-5.377224
6	1	-0.782705	1.015834	1.415039
7	1	1.664316	-0.836626	1.944663
8	1	0.751151	1.800430	3.223758
9	1	0.168311	-1.611257	0.172181
10	6	-1.671567	-0.645713	-0.577783
11	6	-2.078408	-0.339383	-4.451010
12	6	-2.488936	-0.471519	-2.978502
13	6	-1.275757	-0.529760	-2.054683

14	1	-2.301259	-1.534863	-0.453785
15	1	-0.650147	-1.386108	-2.330746
16	1	-2.278199	0.218545	-0.289778
17	1	-0.658926	0.365552	-2.188669
18	1	-1.498162	0.576020	-4.590988
19	1	-1.443815	-1.183892	-4.728670
20	1	-3.121975	0.379740	-2.705859
21	1	-3.088546	-1.376933	-2.851516
22	1	-3.222320	1.734060	-5.928284
23	1	2.249880	0.803767	3.652808
24	6	-3.719803	0.777873	-6.026301
25	1	-4.596162	0.697904	-6.654178
26	7	-3.978233	-1.566643	-5.569894
27	8	-5.007996	-1.560915	-6.203112
28	8	-3.472215	-2.552177	-5.075837

11

E(RM062X) = -406.944387269 A.U.

1	6	-0.468661	-0.779791	0.304728
2	6	-0.143618	0.122028	1.232777
3	6	1.037547	0.030611	2.084282
4	6	1.344502	0.937968	3.009595
5	1	-0.789301	0.985014	1.389664
6	1	1.680556	-0.832978	1.936102
7	1	0.714870	1.805660	3.177194
8	1	0.176288	-1.644672	0.157916
9	6	-1.675402	-0.684832	-0.580351
10	6	-2.083024	-0.275554	-4.420702
11	6	-2.509102	-0.456162	-2.966875
12	6	-1.282354	-0.517744	-2.054006
13	1	-2.286239	-1.589484	-0.476842
14	1	-0.640306	-1.351933	-2.356397
15	1	-2.295512	0.160892	-0.268071
16	1	-0.690086	0.396327	-2.161796
17	1	-1.537727	0.656352	-4.575863
18	1	-1.464737	-1.109621	-4.760541
19	1	-3.154888	0.376153	-2.671158
20	1	-3.090099	-1.379069	-2.862997
21	1	-3.036702	1.638656	-6.054160
22	1	2.227518	0.841849	3.628085
23	6	-3.635896	0.733534	-6.035025
24	1	-4.541119	0.654858	-6.627639
25	7	-3.266000	-0.240812	-5.309267
26	1	-3.847547	-1.080419	-5.298289

12

E(RM062X) = -634.330733964 A.U.

1	6	-0.329020	-0.681959	0.370471
2	6	-0.003803	0.251310	1.266542
3	6	1.148538	0.316478	2.163904

4	6	1.335531	1.247803	3.094222
5	6	-3.433999	-0.312971	-5.181351
6	1	-0.685681	1.084918	1.416824
7	1	0.609355	2.041116	3.213345
8	6	-1.574298	-0.622051	-0.458615
9	6	-2.204727	-0.423546	-4.314086
10	6	-2.522776	-0.511674	-2.816699
11	6	-1.261289	-0.527139	-1.957404
12	1	-2.165708	-1.528264	-0.280776
13	1	-0.628405	-1.372575	-2.250023
14	1	-2.191037	0.229279	-0.154555
15	1	-0.674678	0.378719	-2.142707
16	1	-1.554885	0.437610	-4.494403
17	1	-1.639298	-1.318105	-4.605109
18	1	-3.151896	0.340453	-2.535039
19	1	-3.106112	-1.415641	-2.611322
20	1	-2.948028	1.573016	-5.989120
21	1	-4.534627	0.822412	-6.577753
22	1	2.199936	1.222998	3.741927
23	6	-3.650632	0.747674	-5.954025
24	7	2.224156	-0.710218	2.057808
25	8	2.848518	-0.974911	3.058744
26	8	2.422217	-1.197488	0.966718
27	1	0.333994	-1.520394	0.189499
28	6	-4.401581	-1.464889	-5.118974
29	1	-4.874094	-1.527731	-4.134495
30	1	-5.188585	-1.362403	-5.866236
31	1	-3.882790	-2.413929	-5.286412

13

E(RM062X) = -709.532925924 A.U.

1	6	-0.366673	-0.671733	0.353550
2	6	0.007179	0.246325	1.246307
3	6	1.151621	0.245140	2.156088
4	6	1.386313	1.168693	3.083309
5	6	-3.417393	-0.330612	-5.202978
6	1	-0.622080	1.122568	1.382841
7	1	0.709441	2.006100	3.190410
8	6	-1.596579	-0.539671	-0.489960
9	6	-2.178594	-0.300756	-4.350395
10	6	-2.511775	-0.401666	-2.857344
11	6	-1.261993	-0.477213	-1.985711
12	1	-2.247109	-1.404549	-0.312860
13	1	-0.673619	-1.359738	-2.262240
14	1	-2.161280	0.351488	-0.199717
15	1	-0.623037	0.392430	-2.172550
16	1	-1.640993	0.628183	-4.551323
17	1	-1.503383	-1.117853	-4.630037
18	1	-3.113483	0.468390	-2.574203
19	1	-3.141983	-1.279166	-2.678993
20	1	-3.256394	1.589260	-6.030877
21	1	-4.742175	0.583185	-6.541168
22	1	2.240694	1.094286	3.740448
23	6	-3.823688	0.670601	-5.976272

24	7	2.162324	-0.847021	2.066753
25	8	2.759501	-1.143661	3.075173
26	8	2.340063	-1.352737	0.980464
27	1	0.243849	-1.551995	0.186768
28	8	-4.220410	-1.438170	-5.081536
29	6	-3.567452	-2.691458	-5.170295
30	1	-2.965825	-2.757667	-6.082652
31	1	-2.929360	-2.885833	-4.301734
32	1	-4.353249	-3.443405	-5.203880

TS1

E(RM062X) = -390.505402477 A.U.

1	6	0.006902	0.007660	-0.003551
2	6	0.010386	-0.005775	1.370264
3	6	1.210194	-0.016972	2.102733
4	6	2.436784	-0.002694	1.481517
5	6	1.157139	1.923878	-0.444425
6	1	0.306443	2.484772	-0.069358
7	1	-0.918047	0.198244	1.897915
8	1	1.162445	0.185264	3.168095
9	1	2.579363	-0.471038	0.516247
10	1	0.844289	-0.448561	-0.522190
11	6	-1.247378	0.141321	-0.827571
12	6	1.199941	1.737881	-1.948615
13	6	-0.178310	1.644589	-2.610344
14	6	-0.949928	0.358500	-2.311952
15	1	-1.850434	-0.767257	-0.713899
16	1	-1.889887	0.367651	-2.870672
17	1	-1.855891	0.967435	-0.441952
18	1	-0.374555	-0.497831	-2.684299
19	1	1.796886	0.850676	-2.196933
20	1	1.732934	2.589862	-2.385428
21	1	-0.777361	2.506485	-2.291816
22	1	-0.056648	1.737417	-3.693281
23	1	3.260339	1.715022	-0.162891
24	1	2.355207	2.472203	1.239096
25	1	3.339841	0.111589	2.070523
26	6	2.306308	1.909616	0.317804

TS2

E(RM062X) = -595.003598798 A.U.

1	6	0.033409	0.048155	-0.006510
2	6	0.025094	0.025719	1.357063
3	6	1.226772	-0.002025	2.098513
4	6	2.462867	-0.051427	1.496752
5	6	1.327400	2.103730	-0.209276
6	1	-0.897489	0.250974	1.883459
7	1	1.169869	0.223481	3.157692
8	1	2.593466	-0.550369	0.544980
9	1	0.914495	-0.312911	-0.528735

10	6	-1.170720	0.252884	-0.875396
11	6	1.216168	2.201359	-1.718857
12	6	-0.116047	1.978635	-2.453949
13	6	-0.766594	0.603636	-2.308508
14	1	-1.765851	-0.668059	-0.879467
15	1	-1.651850	0.576328	-2.948992
16	1	-1.803054	1.041514	-0.453266
17	1	-0.087881	-0.172658	-2.683774
18	1	1.957200	1.494843	-2.110174
19	1	1.574281	3.198506	-2.000589
20	1	-0.825813	2.746781	-2.151440
21	1	0.097675	2.142841	-3.514683
22	1	3.371354	1.574050	-0.176398
23	1	3.356329	0.011947	2.106600
24	6	2.518952	1.841084	0.436984
25	7	0.360688	2.823016	0.605624
26	8	-0.599433	3.336341	0.052725
27	8	0.540625	2.854484	1.811852
28	1	2.720742	2.338311	1.372617

TS3

E(RM062X) = -406.945696299 A.U.

1	6	-0.056537	-0.073097	-0.008985
2	6	0.024903	0.074327	1.335582
3	6	1.285080	0.077954	1.999318
4	6	2.501422	0.061363	1.354516
5	1	-0.860982	0.325832	1.910350
6	1	1.282365	0.275667	3.067942
7	1	2.605904	-0.378916	0.369138
8	1	0.816453	-0.451902	-0.535521
9	6	-1.239657	0.170569	-0.879663
10	6	1.384545	1.978089	-1.730093
11	6	-0.030069	1.977994	-2.317832
12	6	-0.812453	0.658983	-2.271540
13	1	-1.780413	-0.777004	-0.997987
14	1	-1.708940	0.779429	-2.882060
15	1	-1.934246	0.867682	-0.402056
16	1	-0.217118	-0.123665	-2.754206
17	1	1.918327	1.054014	-1.971278
18	1	1.955953	2.810902	-2.146700
19	1	-0.609233	2.782883	-1.848604
20	1	0.071205	2.266684	-3.365954
21	1	3.379919	1.826659	0.053162
22	1	3.409224	0.085814	1.946220
23	6	2.419876	1.985427	0.531808
24	1	2.420653	2.521683	1.470063
25	7	1.370760	2.137437	-0.262964
26	1	0.546661	2.580729	0.122924

TS4

E(RM062X) = -634.304456563 A.U.

1	6	0.035942	0.028549	-0.019447
2	6	0.013805	0.021793	1.342894
3	6	1.212428	0.006281	2.083401
4	6	2.457566	0.017170	1.500505
5	6	2.491541	1.853217	0.402099
6	6	1.275746	2.140713	-0.192060
7	1	-0.908082	0.215137	1.879580
8	1	2.597107	-0.535505	0.582668
9	1	0.917286	-0.324454	-0.544739
10	6	-1.194135	0.178663	-0.866608
11	6	1.175072	2.152665	-1.706976
12	6	-0.218720	2.051900	-2.338930
13	6	-0.870102	0.669500	-2.278770
14	1	-1.701493	-0.790860	-0.936707
15	1	-1.790380	0.686674	-2.868426
16	1	-1.900719	0.859679	-0.378848
17	1	-0.206011	-0.057797	-2.761645
18	1	1.822423	1.368114	-2.115703
19	1	1.616796	3.103972	-2.032828
20	1	-0.886113	2.793937	-1.888210
21	1	-0.122247	2.337139	-3.390174
22	1	3.330262	1.574971	-0.228839
23	1	2.748373	2.350402	1.330842
24	1	3.327320	0.139405	2.130715
25	7	1.124627	0.395097	3.496277
26	8	2.144519	0.751538	4.054799
27	8	0.036464	0.336806	4.032710
28	6	0.295582	2.977411	0.589963
29	1	0.413115	2.803852	1.661843
30	1	0.489214	4.040735	0.402535
31	1	-0.743361	2.779357	0.324177

TS5

E(RM062X) = -709.513855897 A.U.

1	6	0.019502	0.044525	0.021533
2	6	0.023667	-0.007151	1.369909
3	6	1.222852	-0.027566	2.138979
4	6	2.477446	0.137546	1.544287
5	6	2.655487	1.919509	0.771669
6	6	1.425459	2.461756	0.382909
7	1	-0.906809	0.093706	1.918033
8	1	2.629789	-0.386075	0.608894
9	1	0.935406	-0.133662	-0.537167
10	6	-1.211314	0.262914	-0.803659
11	6	1.032732	2.702673	-1.049199
12	6	-0.467449	2.665146	-1.394776
13	6	-0.988476	1.314099	-1.897355
14	1	-1.525427	-0.673815	-1.279665
15	1	-1.935509	1.477297	-2.418634
16	1	-2.031488	0.579277	-0.151968
17	1	-0.290804	0.919476	-2.645966
18	1	1.598722	2.019996	-1.687916
19	1	1.411652	3.710509	-1.269124

20	1	-1.044475	2.992658	-0.525740
21	1	-0.638590	3.406143	-2.178750
22	1	3.343230	1.736672	-0.045676
23	1	3.121714	2.344119	1.653461
24	1	3.332331	0.130890	2.207189
25	8	0.589547	3.009108	1.250326
26	6	1.005354	3.318084	2.584513
27	1	1.648399	4.201895	2.558751
28	1	0.092194	3.538250	3.130100
29	1	1.530004	2.496432	3.067038
30	7	1.115837	0.201156	3.545020
31	8	2.125869	0.553748	4.156594
32	8	0.028392	0.065331	4.083865

21

E(RM062X) = -390.613264022 A.U.

1	6	0.239941	0.052205	-0.270620
2	6	0.242289	-0.377606	1.173590
3	6	1.149886	0.029395	2.057286
4	6	2.299290	0.941139	1.710507
5	6	1.089552	1.315266	-0.461605
6	1	0.573698	2.131845	0.066682
7	1	-0.550938	-1.051534	1.489665
8	1	1.076378	-0.302789	3.088964
9	1	3.222478	0.545008	2.144311
10	1	0.698687	-0.750549	-0.870414
11	6	-1.173240	0.271144	-0.821181
12	6	1.166544	1.683855	-1.942409
13	6	-0.233729	1.886790	-2.529062
14	6	-1.121588	0.659417	-2.301302
15	1	-1.772083	-0.634900	-0.685418
16	1	-2.129489	0.847473	-2.680409
17	1	-1.660702	1.066023	-0.243105
18	1	-0.719379	-0.184528	-2.874613
19	1	1.681777	0.880082	-2.484594
20	1	1.767411	2.589531	-2.071517
21	1	-0.696101	2.756297	-2.046851
22	1	-0.170527	2.113710	-3.596568
23	1	2.926552	0.228693	-0.231920
24	1	3.109078	1.968240	-0.019363
25	1	2.137911	1.915891	2.187662
26	6	2.453266	1.119839	0.197300

22

E(RM062X) = -595.103229155 A.U.

1	6	0.248280	0.239308	-0.248422
2	6	0.211213	-0.375406	1.129172
3	6	1.127242	-0.134565	2.060840
4	6	2.287091	0.806439	1.857872
5	6	1.144455	1.483131	-0.292122

6	1	-0.612005	-1.052661	1.337342
7	1	1.037410	-0.604588	3.034863
8	1	3.204873	0.357779	2.246773
9	1	0.775947	-0.472267	-0.902402
10	6	-1.124635	0.448873	-0.902233
11	6	1.279762	2.002757	-1.724007
12	6	-0.093866	2.257882	-2.351457
13	6	-0.963590	0.997077	-2.323631
14	1	-1.644164	-0.513513	-0.929766
15	1	-1.944088	1.207866	-2.756511
16	1	-1.728112	1.126146	-0.296814
17	1	-0.495661	0.231179	-2.954264
18	1	1.815421	1.236794	-2.294959
19	1	1.890842	2.906178	-1.721275
20	1	-0.596644	3.065592	-1.806502
21	1	0.033597	2.608148	-3.378085
22	1	2.926023	0.338082	-0.157034
23	1	2.122833	1.713802	2.452945
24	6	2.483207	1.178523	0.384861
25	7	0.547214	2.648621	0.521201
26	8	-0.490374	2.496871	1.119995
27	8	1.192342	3.675678	0.515627
28	1	3.148039	2.037446	0.282839

23

E(RM062X) = -407.027599578 A.U.

1	6	0.305309	0.129768	-0.318237
2	6	0.254104	-0.420141	1.081109
3	6	1.093802	-0.066668	2.047519
4	6	2.238165	0.885203	1.836135
5	1	-0.544738	-1.128284	1.271642
6	1	0.965053	-0.473178	3.044037
7	1	3.155949	0.464062	2.253025
8	1	0.830081	-0.556102	-0.994228
9	6	-1.073817	0.447922	-0.891900
10	6	1.294716	1.956174	-1.709319
11	6	-0.070572	2.268208	-2.303646
12	6	-0.972703	1.034262	-2.299542
13	1	-1.652206	-0.478069	-0.894888
14	1	-1.964775	1.294949	-2.668641
15	1	-1.591926	1.139340	-0.216244
16	1	-0.566292	0.279765	-2.980775
17	1	1.829867	1.199614	-2.288241
18	1	1.922661	2.843844	-1.626557
19	1	-0.536777	3.080258	-1.734523
20	1	0.079456	2.642868	-3.317367
21	1	2.914020	0.300402	-0.146335
22	1	2.061128	1.818963	2.381150
23	6	2.464080	1.157717	0.357637
24	1	3.085123	2.036852	0.186912
25	7	1.144404	1.392053	-0.322563
26	1	0.626399	2.079375	0.237324

24

E(RM062X) = -634.406714986 A.U.

1	6	0.203269	0.397801	-0.112587
2	6	0.218638	-0.080232	1.310709
3	6	1.207887	0.239814	2.138007
4	6	2.383009	1.109206	1.810383
5	6	2.437831	1.391640	0.302790
6	6	1.051232	1.677895	-0.292623
7	1	-0.579997	-0.719764	1.672613
8	1	3.300270	0.622091	2.146439
9	1	0.702264	-0.390364	-0.699584
10	6	-1.199959	0.541233	-0.708005
11	6	1.159494	1.962159	-1.799538
12	6	-0.209361	2.072638	-2.482909
13	6	-1.090279	0.849069	-2.205250
14	1	-1.759666	-0.386292	-0.555542
15	1	-2.083261	0.995695	-2.636766
16	1	-1.752353	1.331908	-0.189976
17	1	-0.653820	-0.023990	-2.705274
18	1	1.727755	1.147727	-2.266643
19	1	1.735270	2.880425	-1.956929
20	1	-0.723829	2.976738	-2.144125
21	1	-0.069518	2.188243	-3.560683
22	1	2.866877	0.524606	-0.211322
23	1	3.105344	2.238010	0.115612
24	1	2.310574	2.036609	2.387172
25	7	1.146529	-0.263680	3.527617
26	8	2.018004	0.123952	4.278236
27	8	0.249099	-1.015454	3.839794
28	6	0.432922	2.886051	0.428120
29	1	0.227450	2.674573	1.479348
30	1	1.126302	3.731027	0.383307
31	1	-0.504153	3.206726	-0.026293

25

E(RM062X) = -709.612524307 A.U.

1	6	0.203269	0.397801	-0.112587
2	6	0.218638	-0.080232	1.310709
3	6	1.207887	0.239814	2.138007
4	6	2.383009	1.109206	1.810383
5	6	2.437831	1.391640	0.302790
6	6	1.051232	1.677895	-0.292623
7	1	-0.579997	-0.719764	1.672613
8	1	3.300270	0.622091	2.146439
9	1	0.702264	-0.390364	-0.699584
10	6	-1.199959	0.541233	-0.708005
11	6	1.159494	1.962159	-1.799538
12	6	-0.209361	2.072638	-2.482909
13	6	-1.090279	0.849069	-2.205250
14	1	-1.759666	-0.386292	-0.555542
15	1	-2.083261	0.995695	-2.636766
16	1	-1.752353	1.331908	-0.189976

17	1	-0.653820	-0.023990	-2.705274
18	1	1.727755	1.147727	-2.266643
19	1	1.735270	2.880425	-1.956929
20	1	-0.723829	2.976738	-2.144125
21	1	-0.069518	2.188243	-3.560683
22	1	2.866877	0.524606	-0.211322
23	1	3.105344	2.238010	0.115612
24	1	2.310574	2.036609	2.387172
25	7	1.146529	-0.263680	3.527617
26	8	2.018004	0.123952	4.278236
27	8	0.249099	-1.015454	3.839794
28	6	0.432922	2.886051	0.428120
29	1	0.227450	2.674573	1.479348
30	1	1.126302	3.731027	0.383307
31	1	-0.504153	3.206726	-0.026293

M06-2X/6-311G(d,p) gas phase computed total energies and Cartesian coordinates of the stationary points involved in the DA reactions of compounds **14** – **20**.

14

E(RM062X) = 195.257236490 A.U.

1	6	-0.394683	-0.742135	0.284574
2	6	-0.159988	0.094826	1.296698
3	6	1.035521	0.044923	2.130931
4	6	1.275398	0.877494	3.143104
5	1	-0.889426	0.868061	1.531957
6	1	1.760090	-0.729661	1.890374
7	1	0.570572	1.659530	3.405778
8	1	0.346004	-1.508961	0.064254
9	6	-1.611311	-0.713224	-0.585917
10	1	-2.152908	-1.661979	-0.533219
11	1	-2.290917	0.086665	-0.288577
12	1	2.177340	0.804716	3.737324
13	1	-1.339084	-0.561339	-1.634270

15

E(RM062X) = -399.737440194 A.U.

1	6	-0.836870	1.033953	0.907036
2	6	-0.103329	0.035412	1.395988
3	6	1.078336	0.262267	2.231834
4	6	1.264708	1.204039	3.147828
5	1	-0.354540	-0.995525	1.175221
6	1	0.465821	1.897562	3.373854
7	6	-2.077750	0.848437	0.093461
8	1	-1.973163	1.326754	-0.883786
9	1	-2.298094	-0.208595	-0.057595
10	1	2.204768	1.279941	3.675759
11	7	2.194647	-0.712494	2.026071
12	8	3.226872	-0.538237	2.630686
13	8	1.982016	-1.625523	1.259515
14	1	-0.518191	2.058199	1.090225
15	1	-2.935238	1.314516	0.586689

16

E(RM062X) = 117.907559051 A.U.

1	6	-3.454288	-0.305691	-5.282184
2	6	-2.298227	-0.251545	-4.324166
3	1	-1.707254	0.660553	-4.459714
4	1	-1.631108	-1.113811	-4.458349
5	1	-3.136261	1.522704	-6.302199
6	1	-4.595910	0.530940	-6.858348
7	6	-3.743480	0.626195	-6.191033

8	1	-2.644645	-0.282349	-3.282250
9	1	-4.093333	-1.187140	-5.209080

17

E(RM062X) = -322.357058420 A.U.

1	6	-3.389956	-0.213561	-5.309786
2	6	-3.644305	0.746074	-6.183984
3	1	-3.958432	-1.133031	-5.318888
4	7	-4.738653	0.497523	-7.167849
5	8	-5.334948	-0.553486	-7.119901
6	8	-4.955161	1.387473	-7.961658
7	6	-2.974489	2.071039	-6.326283
8	1	-2.533794	2.175736	-7.318425
9	1	-3.693825	2.881743	-6.204263
10	1	-2.194591	2.161895	-5.571768
11	1	-2.605767	-0.081100	-4.576129

18

E(RM062X) = -134.261883158 A.U.

1	6	-2.074491	-0.272494	-4.444303
2	1	-1.538180	0.667721	-4.546743
3	1	-1.451623	-1.106689	-4.765500
4	1	-3.029776	1.633297	-6.071336
5	6	-3.634730	0.732298	-6.036967
6	1	-4.542093	0.649216	-6.625977
7	7	-3.272841	-0.234793	-5.296812
8	1	-3.858630	-1.070984	-5.281901
9	1	-2.392227	-0.422794	-3.413057

19

E(RM062X) = -157.179385878 A.U.

1	6	-3.468958	-0.331050	-5.276314
2	6	-2.300642	-0.240774	-4.333514
3	1	-1.728603	0.674359	-4.486909
4	1	-1.631260	-1.096002	-4.468427
5	1	-3.109566	1.497422	-6.262716
6	1	-4.578455	0.534629	-6.846386
7	6	-3.732129	0.614176	-6.173247
8	1	-2.642678	-0.266988	-3.294290
9	6	-4.319393	-1.563441	-5.135138
10	1	-3.719425	-2.464702	-5.294609
11	1	-4.731312	-1.631959	-4.123542
12	1	-5.145419	-1.566824	-5.846407

20

E(RM062X) = -232.380259031 A.U.

1	6	-3.322741	-0.286394	-5.451317
2	6	-2.113028	-0.294308	-4.561265
3	1	-1.799339	0.727118	-4.354186
4	1	-1.288199	-0.824720	-5.045247
5	1	-3.605640	1.790458	-5.535869
6	1	-4.822674	0.743082	-6.484759
7	6	-3.958541	0.814875	-5.838210
8	1	-2.313089	-0.789366	-3.607661
9	8	-3.744714	-1.492756	-5.944837
10	6	-3.679077	-2.600374	-5.067862
11	1	-4.131280	-3.435064	-5.599463
12	1	-2.647821	-2.861538	-4.812169
13	1	-4.244255	-2.413086	-4.148615

TS6

E(RM062X) = -313.094312876 A.U.

1	6	0.033736	0.025325	-0.003580
2	6	0.020307	0.000345	1.372516
3	6	1.195404	-0.018032	2.137059
4	6	2.440435	0.038070	1.545296
5	6	1.370604	1.863388	-0.462083
6	1	0.457542	2.432882	-0.335274
7	1	-0.920298	0.193411	1.882000
8	1	1.116401	0.163168	3.204159
9	1	2.615449	-0.430479	0.584137
10	1	0.859729	-0.446231	-0.524516
11	6	-1.227364	0.200413	-0.801716
12	6	1.757600	1.492737	-1.871434
13	1	-1.566866	-0.752927	-1.217570
14	1	-2.028349	0.603896	-0.179404
15	1	0.936659	1.036258	-2.429105
16	1	2.593557	0.786564	-1.864409
17	1	3.382286	1.822246	0.237453
18	1	2.165141	2.517782	1.412675
19	1	3.323949	0.131242	2.167314
20	6	2.340084	1.930609	0.521164
21	1	2.080421	2.374725	-2.432806
22	1	-1.081943	0.882070	-1.644628

TS7

E(RM062X) = -517.596762249 A.U.

1	6	0.159232	0.050406	-0.087751
2	6	0.077285	-0.058735	1.271529
3	6	1.207668	-0.100625	2.108009
4	6	2.499948	-0.041066	1.615193
5	6	1.664615	2.131285	-0.037917
6	1	-0.893044	0.073987	1.739536

7	1	1.050572	0.022994	3.174115
8	1	2.722671	-0.477797	0.648477
9	1	1.079038	-0.217739	-0.595546
10	6	-1.035571	0.302620	-0.949355
11	6	1.887648	2.162391	-1.517933
12	1	-1.951377	0.293066	-0.357153
13	1	-0.969615	1.288253	-1.423786
14	1	2.742494	1.529159	-1.763176
15	1	2.104427	3.180281	-1.853210
16	1	3.649282	1.707665	0.525914
17	1	3.326668	-0.060487	2.316172
18	6	2.643455	1.851937	0.903502
19	7	0.499897	2.824989	0.448979
20	8	-0.259548	3.306956	-0.381732
21	8	0.320877	2.888521	1.657368
22	1	2.558585	2.333456	1.866651
23	1	1.018924	1.819425	-2.079174
24	1	-1.116473	-0.444121	-1.743031

TS8

E(RM062X) = -329.534894500 A.U.

1	6	-0.251090	-0.040465	-0.026131
2	6	-0.030405	0.095730	1.304478
3	6	1.269787	0.124840	1.884949
4	6	2.463286	0.143728	1.189924
5	1	-0.878817	0.232035	1.967115
6	1	1.317955	0.269450	2.961589
7	1	2.519227	-0.260891	0.184631
8	1	0.586460	-0.261151	-0.683331
9	6	-1.591758	0.000013	-0.664579
10	6	1.837709	1.996133	-1.758552
11	1	-1.810101	-0.975995	-1.110643
12	1	-2.378988	0.249330	0.045197
13	1	2.464438	1.106382	-1.847406
14	1	2.362306	2.844708	-2.200934
15	1	3.468185	1.982232	0.314549
16	1	3.384522	0.082581	1.758113
17	6	2.428721	2.044393	0.622491
18	1	2.251281	2.556158	1.558947
19	7	1.540108	2.246101	-0.351564
20	1	0.649795	2.661542	-0.119626
21	1	0.910604	1.827130	-2.303281
22	1	-1.608582	0.718141	-1.490246

TS9

E(RM062X) = -556.894262573 A.U.

1	6	0.113687	0.081255	-0.014839
2	6	0.051715	0.041200	1.344624
3	6	1.208653	-0.013729	2.146459
4	6	2.488193	0.041118	1.620928

5	6	2.664493	1.869762	0.813897
6	6	1.641716	2.179098	-0.073850
7	1	-0.894335	0.203368	1.849094
8	1	2.665821	-0.486167	0.692674
9	1	1.013519	-0.234886	-0.529808
10	6	-1.104066	0.278373	-0.863232
11	6	1.876776	2.057560	-1.553618
12	1	-1.348237	-0.631928	-1.418545
13	1	-1.967660	0.549996	-0.254099
14	1	2.626072	1.295600	-1.779569
15	1	2.247333	3.012733	-1.944492
16	1	3.645665	1.665197	0.395845
17	1	2.673948	2.366014	1.779117
18	1	3.318481	0.067347	2.313691
19	7	1.051321	0.302636	3.562294
20	8	2.042740	0.640345	4.185688
21	8	-0.059773	0.211645	4.047583
22	1	0.961752	1.825550	-2.102577
23	1	-0.953859	1.066844	-1.608207
24	6	0.514848	3.065778	0.378430
25	1	0.813995	4.116854	0.279520
26	1	-0.388159	2.923845	-0.219154
27	1	0.276120	2.887573	1.429052

TS10

E(RM062X) = -632.106279490 A.U.

1	6	0.112936	0.210428	0.113024
2	6	0.080816	0.159536	1.453087
3	6	1.233284	0.008516	2.310634
4	6	2.555875	-0.020576	1.844461
5	6	3.326265	1.722977	1.729524
6	6	2.398756	2.513816	1.055103
7	1	-0.868640	0.270548	1.966429
8	1	2.690205	-0.366202	0.825932
9	1	1.053694	0.077874	-0.417240
10	6	-1.096373	0.435141	-0.742475
11	6	2.453749	2.747401	-0.420695
12	1	-1.251084	-0.391239	-1.442570
13	1	-1.994071	0.535714	-0.130648
14	1	3.027592	1.965505	-0.917205
15	1	2.941467	3.708129	-0.614566
16	1	4.235506	1.524803	1.174404
17	1	3.464800	1.858770	2.795756
18	1	3.297824	-0.389260	2.541051
19	8	1.355324	3.076696	1.606686
20	6	1.109331	3.123879	3.022443
21	1	0.122936	2.692951	3.191658
22	1	1.843263	2.567158	3.598745
23	1	1.116102	4.175388	3.305711
24	7	1.065146	0.208120	3.694405
25	8	2.079230	0.333076	4.398020
26	8	-0.068534	0.317725	4.157148
27	1	1.444375	2.788556	-0.828033

29

E(RM062X) = -313.199448349 A.U.

1	6	0.042329	0.380623	-0.382142
2	6	0.449065	-0.462557	0.802310
3	6	1.493931	-0.203914	1.585125
4	6	2.404622	0.976690	1.383804
5	6	1.170325	1.344871	-0.797213
6	1	0.731662	2.128663	-1.424853
7	1	-0.175944	-1.325735	1.018238
8	1	1.717083	-0.871303	2.412643
9	1	3.367835	0.629307	0.989370
10	1	-0.141047	-0.300940	-1.222989
11	6	-1.277200	1.108133	-0.092765
12	6	2.235945	0.615330	-1.618550
13	1	-2.052991	0.402743	0.214390
14	1	-1.152358	1.832594	0.715380
15	1	1.821957	0.266889	-2.567621
16	1	2.613383	-0.258777	-1.081370
17	1	2.532146	2.745767	0.138888
18	1	1.002478	2.556667	0.983229
19	1	2.629252	1.436284	2.350693
20	6	1.782009	2.010503	0.443322
21	1	3.081978	1.271560	-1.839607
22	1	-1.631164	1.637604	-0.980829

30

E(RM062X) = -517.689618395 A.U.

1	6	0.415670	0.368761	-0.260443
2	6	0.462593	-0.469847	0.992334
3	6	1.313555	-0.306260	1.998045
4	6	2.368411	0.758094	2.021120
5	6	1.341784	1.610215	-0.212527
6	1	-0.284532	-1.257228	1.046469
7	1	1.240738	-0.954782	2.865373
8	1	3.311520	0.333273	2.378055
9	1	0.810711	-0.238668	-1.085091
10	6	-1.037370	0.721944	-0.607945
11	6	1.734683	2.060384	-1.612899
12	1	-1.511002	1.249621	0.223311
13	1	-1.103965	1.348500	-1.498846
14	1	2.356355	1.283542	-2.061121
15	1	2.304710	2.989180	-1.576431
16	1	3.199660	0.657646	0.049245
17	1	2.080289	1.530373	2.738008
18	6	2.598127	1.359462	0.635146
19	7	0.530358	2.734679	0.436133
20	8	0.145077	3.642288	-0.265081
21	8	0.279011	2.625287	1.616270
22	1	3.172192	2.288049	0.701810
23	1	0.862835	2.224640	-2.243846
24	1	-1.603752	-0.192333	-0.791408

31

E(RM062X) = -329.606481166 A.U.

1	6	0.298530	0.139939	-0.341760
2	6	0.279321	-0.442670	1.048838
3	6	1.100613	-0.088090	2.029459
4	6	2.215288	0.900452	1.842456
5	1	-0.495155	-1.181695	1.220435
6	1	0.979090	-0.525750	3.013454
7	1	3.138847	0.511338	2.277339
8	1	0.802729	-0.535184	-1.041635
9	6	-1.112710	0.443204	-0.831945
10	6	1.370522	1.955988	-1.707353
11	1	-1.706789	-0.467020	-0.746685
12	1	-1.589476	1.203563	-0.207209
13	1	1.890799	1.202617	-2.297547
14	1	1.982373	2.851935	-1.623868
15	1	2.912335	0.304054	-0.125828
16	1	1.999075	1.831719	2.377637
17	6	2.458742	1.165378	0.368439
18	1	3.083185	2.042308	0.201179
19	7	1.147635	1.401397	-0.339540
20	1	0.626529	2.095098	0.207567
21	1	0.416713	2.202469	-2.164499
22	1	-1.144548	0.751877	-1.875742

32

E(RM062X) = -556.985026278 A.U.

1	6	0.313089	0.560900	0.029431
2	6	0.162775	0.155974	1.470459
3	6	1.259939	0.097934	2.215972
4	6	2.578071	0.542640	1.672066
5	6	2.333395	1.875481	0.949126
6	6	1.249882	1.808961	-0.159785
7	1	-0.799845	-0.132984	1.877395
8	1	2.965078	-0.211714	0.976419
9	1	0.845819	-0.274451	-0.448234
10	6	-1.047297	0.687319	-0.656785
11	6	1.933972	1.680899	-1.526164
12	1	-1.575810	-0.267311	-0.618446
13	1	-1.670636	1.435813	-0.163328
14	1	2.623141	0.831244	-1.538224
15	1	2.506777	2.583513	-1.753756
16	1	3.267397	2.246049	0.518650
17	1	2.026728	2.598488	1.710192
18	1	3.306372	0.654610	2.472453
19	7	1.168749	-0.365543	3.612010
20	8	2.206175	-0.438993	4.236873
21	8	0.075664	-0.651378	4.049918
22	1	1.204759	1.535772	-2.327662

23	1	-0.944110	0.969370	-1.705986
24	6	0.443728	3.112028	-0.115308
25	1	1.124721	3.966912	-0.141445
26	1	-0.235934	3.202086	-0.965314
27	1	-0.141839	3.179361	0.806378

33

E(RM062X) = -632.198026011 A.U.

1	6	0.313089	0.560900	0.029431
2	6	0.162775	0.155974	1.470459
3	6	1.259939	0.097934	2.215972
4	6	2.578071	0.542640	1.672066
5	6	2.333395	1.875481	0.949126
6	6	1.249882	1.808961	-0.159785
7	1	-0.799845	-0.132984	1.877395
8	1	2.965078	-0.211714	0.976419
9	1	0.845819	-0.274451	-0.448234
10	6	-1.047297	0.687319	-0.656785
11	6	1.933972	1.680899	-1.526164
12	1	-1.575810	-0.267311	-0.618446
13	1	-1.670636	1.435813	-0.163328
14	1	2.623141	0.831244	-1.538224
15	1	2.506777	2.583513	-1.753756
16	1	3.267397	2.246049	0.518650
17	1	2.026728	2.598488	1.710192
18	1	3.306372	0.654610	2.472453
19	7	1.168749	-0.365543	3.612010
20	8	2.206175	-0.438993	4.236873
21	8	0.075664	-0.651378	4.049918
22	1	1.204759	1.535772	-2.327662
23	1	-0.944110	0.969370	-1.705986
24	6	0.443728	3.112028	-0.115308
25	1	1.124721	3.966912	-0.141445
26	1	-0.235934	3.202086	-0.965314
27	1	-0.141839	3.179361	0.806378
